# Brain Disorders and Chemical Pollutants: A Gap Junction Link? ^†^

**DOI:** 10.3390/biom11010051

**Published:** 2020-12-31

**Authors:** Marc Mesnil, Norah Defamie, Christian Naus, Denis Sarrouilhe

**Affiliations:** 1 Laboratoire STIM, ERL7003 CNRS-Université de Poitiers, 1 rue G. Bonnet–TSA 51 106, 86073 Poitiers, France; marc.mesnil@univ-poitiers.fr (M.M.); norah.defamie@univ-poitiers.fr (N.D.); 2 Faculty of Medicine, Department of Cellular & Physiological Sciences, Life Sciences Institute, University of British Columbia, 2350 Health Sciences Mall, Vancouver, BC V6T1Z3, Canada; ccnaus@gmail.com; 3 Laboratoire de Physiologie Humaine, Faculté de Médecine et Pharmacie, 6 rue de La Milétrie, bât D1, TSA 51115, 86073 Poitiers, France

**Keywords:** Alzheimer’s disease, attention deficit hyperactivity disorders, autism spectrum disorders, blood brain barrier, Brain, connexin, epilepsy, gap junction, glioma, major depressive disorders, migraines, Parkinson’s disease, pesticides, pollutants

## Abstract

The incidence of brain pathologies has increased during last decades. Better diagnosis (autism spectrum disorders) and longer life expectancy (Parkinson’s disease, Alzheimer’s disease) partly explain this increase, while emerging data suggest pollutant exposures as a possible but still underestimated cause of major brain disorders. Taking into account that the brain parenchyma is rich in gap junctions and that most pollutants inhibit their function; brain disorders might be the consequence of gap-junctional alterations due to long-term exposures to pollutants. In this article, this hypothesis is addressed through three complementary aspects: (1) the gap-junctional organization and connexin expression in brain parenchyma and their function; (2) the effect of major pollutants (pesticides, bisphenol A, phthalates, heavy metals, airborne particles, etc.) on gap-junctional and connexin functions; (3) a description of the major brain disorders categorized as neurodevelopmental (autism spectrum disorders, attention deficit hyperactivity disorders, epilepsy), neurobehavioral (migraines, major depressive disorders), neurodegenerative (Parkinson’s and Alzheimer’s diseases) and cancers (glioma), in which both connexin dysfunction and pollutant involvement have been described. Based on these different aspects, the possible involvement of pollutant-inhibited gap junctions in brain disorders is discussed for prenatal and postnatal exposures.

## 1. Introduction

In the last few decades, brain pathologies have become more prevalent in our societies (Table 1) [1]. For some of these pathologies, the increase in their prevalence is probably the consequence of a better diagnosis [autism spectrum disorders, (ASD)] while others, considered as degenerative, are more commonly associated with aging [Parkinson’s (PD) and Alzheimer’s diseases (AD)] [1]. For some of these degenerative pathologies, the incidence increase is most prevalent in high-income countries where life expectancy is the highest. For instance, in the US, the annual incidence rate of AD increases from 1% among people aged 65 years to 8% for people aged 85 years and older. Based on the projected ageing of the population, these numbers will triple in the next 30 years resulting in an increase of nearly 80% in total societal costs per adult [2]. This situation has reached a point where the proportion of deaths related to AD went up by 146.2% between 2000 and 2018, while the overall death rate from stroke, HIV and cardio-vascular diseases decreased. Globally, direct and indirect costs for healthcare related to AD are estimated at nearly USD 500 billion annually [2,3]. Taken together, all brain pathologies, therefore, represent a strong challenge for next few years, in terms of health care and social impact. 

Depending on their etiology, diagnosis and evolution, these brain pathologies can be classified into three major categories of disorders (Table 1), as neurodevelopmental [ASD, attention deficit hyperactivity disorders (ADHD), epilepsy], neurodegenerative (PD and AD) and neurobehavioral [migraines, major depressive disorders (MDD)]. Brain cancer can be included in this list as a 4th category since it cannot be classified as either a neurodevelopmental, neurodegenerative or neurobehavioral disorder (Table 1). Even if still rare (less than 2% of cancer cases), brain cancer incidence has increased in high income countries similar to other brain pathologies [4]. The recent rise in incidence of these various brain pathologies [1,4] leads one to question their possible common etiology. 

Part of the answer to this question might be in distinct characteristics of the brain. Contrary to other organs, human brain development still progresses for almost two decades after birth, especially during adolescent years. Even later in life, contrary to earlier held views, the brain is not a “static” organ. This brain plasticity permits humans to adapt their behavior to their environment throughout their life [5]. Therefore, during intra-uterine and postnatal periods of life (childhood, adolescence), neuronal connections are modulated under environmental cues and stimulation (education, psychological traumatisms, etc.) and are maintained during the rest of life. Such connections are the consequences of cellular plasticity, which depends on synaptic activity and on the presence of stem cells able to replace damaged brain cells including neurons, astrocytes, and oligodendrocytes. Due to its multiple functions crucial for survival of the entire organism, the brain is highly protected against mechanical and chemical trauma by the skull externally and the blood–brain barrier (BBB) internally. However, despite this high level of protection, the brain can be reached and exposed to compounds able to cross the BBB. This leads, for instance, to drug addictions and possibly to the higher prevalence of brain pathologies affecting humans from birth (ASD, ADHD, epilepsy) to old age (PD and AD). 

In order to assure coordinated brain activity, major brain cells (neurons, astrocytes, oligodendrocytes) are tightly connected through a dense intercellular network. Among such intercellular interactions, gap junctions (GJs) play important roles necessary to brain activity by permitting an efficient transfer of information between neurons through electrical synapses, neurotransmitter and ionic buffering of chemical synapses, axonal myelination, neuronal metabolism through astrocytes, etc. Consequently, the brain is one of the organs abundantly supplied by diversity of GJ-proteins [connexins (Cxs)]. Therefore, considering the sensitivity of Cxs and GJ functions to a wide range of chemical compounds, it is possible that chronic exposures to BBB-permeable pollutants disturb the gap-junctional intercellular communication (GJIC) network in brain. Such GJIC disturbances could then perturb brain physiology (synaptic plasticity, memory storage, behavior, etc.) and explain the rising incidence of brain pathologies. 

The aim of the present review is to evaluate whether chemical pollutants can induce neuropathologies through their actions on GJIC and Cxs. In order to argue about such a possibility, this review is made of three major parts reviewing (1) the physiological roles of GJs and Cxs in the brain, (2) the effect of chemical pollutants on Cx functions and, (3) the Cx and environmental involvements in major brain pathologies cited above (Table 1).

## 2. Gap Junction and Connexin Roles in Brain

In the context of animal evolution, the brain was formed through the cephalisation process. As the anterior part of the central nervous system (CNS), it results from both an increased number of neurons and a particular cellular organization. The adult human brain weighs approximately 1.5 kg and, proportional to the body size, its volume is the highest of all animal species. As a complex organ, the brain is rich in GJs and probably exhibits the widest range of Cx expression in the body with most of the 21 Cx isoforms present (Cx26, Cx29, Cx30, Cx30.2, Cx31.1, Cx31.9, Cx32, Cx36, Cx37, Cx40, Cx43, Cx45, Cx46, Cx47, Cx57) [18,19,20,21,22,23,24]. Paradoxically, while the brain is highly complex in its functional capacities and tissue organization, it only contains a few different cell types expressing, for most of them, several Cx isoforms. Before being stabilized in each differentiated cell type, the Cx expression profile varies during embryogenesis and reveals that Cxs are involved both in brain formation and in many physiological aspects needed for its functions (Table 2). 

### 2.1. Brain Development and Connexin Expression

The brain is the result of an embryonic process starting from the 3rd gestational week to undergo the four following principal phases of development: (1) dorsal induction (primary and secondary neurulations), (2) ventral induction (forebrain patterning), (3) neuronal proliferation and migration, and (4) myelination. 

Thus, during the 3rd week of embryogenesis, initiation of the CNS evolves with the development of the notochordal process (neuroectoderm induction). This derivative of primitive ectoderm rapidly grows in length to be converted, within 20 days, from a hollow tube to the notochord. Then, the notochord, in relation with the axial mesoderm, induces the neural plate (primary neurulation). At this stage, the neuroepithelium of the neural plate begins the formation of the brain and spinal cord by appearing at the cranial end of the embryo to differentiate craniocaudally. At the beginning of the 4th week, the neural plate is composed of a broad cranial portion (foetal brain) and a narrow caudal portion (spinal cord). Then, the foetal brain pursues its own particular and complex evolution, which corresponds, for the next 5 weeks, to the proencephalic and hemispheric formation. 

From the 10th week, neuronal proliferation continues for at least 10 weeks. Soon (12th week), neuronal migration takes place concomitant with neuronal proliferation up to the 24th week. This neuronal proliferation (neurogenesis) within the cerebral hemispheres arises from neural precursor cells (NPCs) which are either “type 1 stem-like cells” (nestin^+^, GFAP^+^) or “type 2a progenitors” (nestin^+^, GFAP^-^). These self-renewing and multipotential cells are able to differentiate either into neurons or glial cells. Neurogenesis starts when those cells differentiate in “type 2b progenitor” cells (nestin^+^, doublecortin^+^) to become “type 3 neuroblasts” (doublecortin^+^) capable of migrating to form the cortical layers [24,99,100,101,102,103,104]. Thereafter, those post-mitotic neurons complete their differentiation by extending axon and dendrites from their cell body. Neuronal proliferation and migration continue after birth for several months (postnatal neurogenesis) or even years. Synaptogenesis accompanies this phenomenon up to puberty, and even probably later. Apparently, NPCs, which are at the origin of neurogenesis, are differently located during embryogenesis. For instance, those from the ventricular zone (VZ) are at the site of origin of cortical glutamatergic pyramidal neurons, while those from the ganglionic eminence form GABAergic neurons. After neuronal proliferation and differentiation, programmed neuronal cell death also takes place from the 28th week up to at least the first postnatal week. During all this neuronal organization, gliogenesis (from the 20–24th weeks) and myelination (from the 26–28th weeks) complete the cellular brain organization up to 2–3 postnatal years and maybe more. Finally, as brain tissue grows and differentiates, angiogenesis forms the particular blood vessel network necessary for food and oxygen supply to neurons and other brain cells. Importantly, at the time of birth, the BBB is not finalized due to the incomplete coverage of blood vessels by astrocytic endfeet. Such a process seems to be completed at postnatal day 10 (P10) and the BBB is considered being mature at P20 [105].

**Figure 1:**
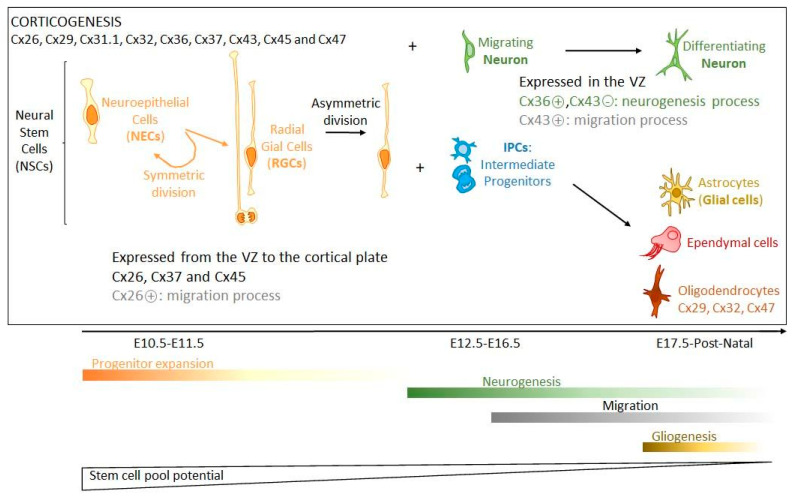
Corticogenesis. During early cortical development, neural stem cells (NSCs) predominantly undergo symmetric divisions to expand the NSC pool (Progenitor expansion phase). This pool includes neuroepithelial, radial glial and intermediate progenitor cells. Neuroepithelial cells (NECs) become radial glial cells (RGCs), which by asymmetric divisions generate RGC and intermediate progenitors (IPCs) or neurons, both migrating and differentiating (Neurogenesis phase). At later stages of development, NSCs generate the other cell types of the brain including astrocytes, oligodendrocytes, or ependymal cells (Gliogenesis phase). During cortical development, Cx37 and Cx45 are largely distributed from the VZ to the cortical plate, whereas Cx36 and Cx43 are highly expressed in the VZ and less in the cortical plate. While Cx43 is a negative modulator of neuronal differentiation, Cx36 activates this process (in green). Cx26 and Cx43 are involved in neocortical neuronal migration (in grey). The differentiation of oligodendrocytes is accompanied by the expression of specific Cxs (Cx29, Cx32, Cx47) that are involved in the postnatal myelination process. The time scale of these phases of corticogenesis (E: Embryogenesis days) is from mouse [136].

Cxs and GJIC play important roles in organs during development [106]; this is also the case for brain formation and cortical development (Figure 1). In rodents, at least 9 Cx isoforms are highly expressed in the embryonic cerebral cortex (Cx26, Cx29, Cx31.1, Cx32, Cx36, Cx37, Cx43, Cx45 and Cx47) [24,107,108], and during cortical development, each Cx isoform has a distinct spatial and temporal pattern of expression [107,109,110,111], which might have functional significance as each Cx isoform has distinct permeability and regulation properties [112]. Cx26, Cx37 and Cx45 are largely distributed from the VZ to the cortical plate, whereas Cx36 and Cx43 are highly expressed in the VZ and less in the cortical plate [108,113]. Cx43 is widely expressed in embryonic brain before being restricted mainly to astrocytes as the CNS matures. While Cx43 is a negative modulator of neuronal differentiation through its carboxyl terminus (C-ter), Cx36 activates this process [114,115]. This is consistent with Cx36 expression in the VZ during the first wave of neurogenesis with, to a lesser extent, Cx26 [108]. Cx36 expression is maintained during the period of neuronal differentiation before being dramatically reduced during postnatal maturation. It is then restricted to only a few neuron populations (interneurons of hippocampus, thalamus, and sparcely in neocortex) where it forms electrical synapses, while most other neurons are connected by chemical synapses [108,109,116,117,118]. Interestingly, two other Cxs (Cx26 and Cx43) are involved in neocortical neuronal migration, which seems to be mainly the consequence of mechanisms driven by the C-ter part of Cx43, independently from its channel function, but depending on its phosphorylation status [113,119,120,121]. In this process, astrocytes participate in the migration of neurons by orientating them [113], and become involved in synaptogenesis, contributing eventually to the formation of tripartite synapses. The differentiation of oligodendrocytes is accompanied by the expression of specific Cxs (Cx29, Cx32, Cx47), which are involved in the postnatal myelination process [63,69,122].

During brain development, the presence of Cxs leads to the establishment of GJIC, which is important to coordinate differentiation of NPC clusters. This is particularly true in the rodent embryonic CNS where spatio-temporal patterns necessary for formation of functional domains have to be synchronized in their behaviour [106,123,124]. Moreover, GJIC probably permits NPCs to exchange signaling messages with adjacent cells to direct them towards a particular differentiation lineage (neuronal or glial) [24,125]. Such GJIC compartmentalization is influenced further by changes of Cx expression over time depending on the differentiation process. In parallel to the establishment of GJIC, Cxs are also involved in brain development via their hemichannel (HC) activity. For instance, during corticogenesis, HC-mediated Ca^2+^ waves increase in number, amplitude, and distance [126]. Moreover, in the early postnatal neocortex, dendritic GJs mediate the propagation of IP_3_ and Ca^2+^ waves. This process is thought to be at the origin of functional regionalization by dividing the immature neocortex into columnar patches of coordinated activity [123,127,128,129].

In summary, Cxs are actively involved in brain formation, which largely extends the classical period of embryogenesis. The numerous Cx isoforms are expressed differently, in a spatio-temporal pattern modulating cell proliferation, differentiation and migration. All these processes are mediated differently by Cxs, which establish either GJIC-forming communicating domains directed towards particular differentiation lineages, paracrine/autocrine communication through HCs or particular intracellular protein interactions. Activated P2X7 receptors are involved in apoptosis and in the clearance of apoptotic cells during neurogenesis [130,131,132,133,134]. Even if not completely defined yet, as pannexin (Panx) channels, Cx HCs could participate in such activation by releasing ATP [24]. Finally, Cx protein interactions can take place either outside the cells like extracellular parts of Cx43 interacting with astrocytes during neuronal migration, or inside the cells by controlling particular signaling pathways. Knowing that several Cxs can be expressed in the same cell type, the events can be controlled in a very subtle way depending on their respective abundance, since different Cxs may have opposite effects in a same cell type (eg., Cx43 and Cx36 in neuronal differentiation) [24,119,135]. 

### 2.2. Adult Brain Organization and Connexin Involvement

At term, adult brain tissue is made of a few cell types contributing to different functional compartments such as neuronal, glial, and vascular compartments. The neuronal compartment is only made of neurons interacting between each other either through abundant chemical or less frequent electrical synapses. This compartment is complex with regard to different types of neurons (projection neurons, interneurons, etc.) presenting a high variety in spatial and temporal neurotransmitter release and receptor activation. The glial compartment is the most complex in terms of cell types and organization. Even if glial cells are smaller than neurons, they are 10 times more numerous and constitute at least 50% of the total brain cells [137,138]. The glial compartment is composed of macroglia (astrocytes, oligodendrocytes, ependymocytes) and microglia. The most abundant glial cells are astrocytes whose ramifications are, either, in contact with neuronal synapses (tripartite synapses), oligodendrocytes or cover environing blood capillaries by their endfeet, which are part of the BBB. Astrocytes control the extracellular interstitial space composition by buffering K^+^ ions and clearing neurotransmitters secreted by neuronal activity. They also secrete gliotransmitters, which can modulate neuronal activity [139]. Oligodendrocytes are myelinating cells whose ramifications surround axons of neurons by forming myelin, which facilitates the propagation of action potentials along axons; myelinated axons constitute the white matter of the CNS. Ependymocytes line the ventricular cavities of brain. They constitute a permeable barrier between the cerebrospinal fluid (CSF) and the interstitial liquid surrounding cells of the CNS. Some of these cells are ciliated and permit the circulation of the CSF [140,141,142,143]. Microglia control inflammation and can differentiate into macrophagocytes in the presence of microorganisms or damaged neurons to phagocytose them. Their role is to protect brain tissue since cells of the immune system cannot normally access the CNS. The vascular compartment is made of endothelial cells, which line brain capillaries. The apical face of endothelial cells is in contact with blood whereas their basal face is in contact, for some of them, with pericytes whose contracting capacity regulates blood flow. The vascular compartment is separated physically from the glial compartment by the basal membrane. Therefore, anatomically, the glial compartment is localized between vascular and neuronal compartments. 

Due to the limited extracellular space (20%) of CNS, these different cell types are at high density and extensively interconnected between themselves. Such density and interconnection facilitate neuron function by stabilizing their immediate extracellular environment (astrocytes, oligodendrocytes), bringing nutriments (astrocytes), protecting them (microglia) and increasing the speed of their communication (oligodendrocytes). Most of these very different actions have to be highly coordinated and, therefore, all cells are highly communicating between each other through direct (GJs) or short distance (autocrine/paracrine) (HCs) communication. Interestingly, both types of intercellular communication (direct or autocrine/paracrine), are mediated by the presence of Cxs. This explains why Cxs are so abundant in the brain parenchyma (Table 2).

#### 2.2.1. Gap-Junction Compartments in Brain

**Figure 2:**
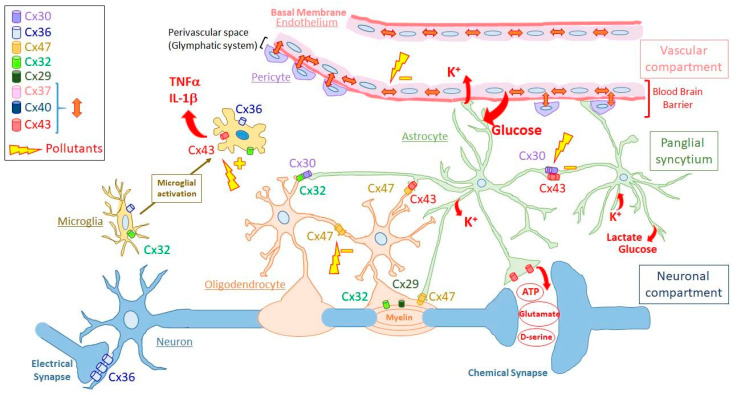
Schematic representation of the organization and connexin composition of the gap-junction compartments in the human adult brain. The different gap-junction compartments are indicated on the right side of the figure (vascular compartment, panglial syncytium, neuronal compartment). Principal cell types are presented for each gap-junction compartment (vascular compartment: endothelial cells or endothelium with pericytes; panglial syncytium: astrocytes in green and oligodendrocytes with myelin surrounding axons of neurons; neuronal compartment: neurons connected by chemical or electrical synapses). For clarity, pericytes are not enveloped by basement membrane. Microglia are located apart since they do not seem to establish permanent gap junctions between microglial cells or with cells of the different gap-junction compartments. Microglial cells are presented in non-activated and activated states through microglial activation. The connexin isoforms expressed in the different cell types are indicated according to the code color inserted in the left side of the figure. For clarity, connexin hemichannels and gap junctions are both presented as cylinders. Cylinders present between cells are gap junctions made of the indicated connexins, while those located in nonintercellular areas are hemichannels. Cylinders inside myelin represent reflexive gap junctions between the concentrical layers of myelin around neuronal axons. The red double arrows in the vascular compartment represent gap junctions made of either Cx43, Cx37 or Cx40 between endothelial cells and between endothelial cells and pericytes. Single red arrows represent ions (K^+^) and molecules passing through connexin channels, which are either gap junctions (transfer of K^+^ and glucose uptaken from extracellular space to be released in blood or close to neurons, respectively) or hemichannels (release of pro-inflammatory cytokines like TNF-α and IL-1β by activated microglia; ATP, D-serine or glutamate released by astrocytes). Yellow lightning bolts represents possible effects of pollutants as either, inhibitors (−) of gap-junctional intercellular communication or indirect activators (+) of connexin hemichannels (See Discussion part for details). For clarity, ependymocytes and stem cell niches are not shown and only two astrocytic endfeet are presented as constituents of the blood–brain barrier. At the level of the blood-brain barrier, the perivascular space delimited by the astrocytic endfeet is part of the so-called glymphatic system involved in draining interstitial fluid and removing metabolic wastes.

Interestingly, the functional compartments presented above can be viewed as each corresponding to a GJ compartment. Indeed, each of these functional compartments can be defined as a directly communicating network in which GJs permit the free diffusion of ions and small hydrophilic molecules between cytosols of adjacent cells belonging to the same functional compartment. Therefore, GJIC defines at least three different GJ compartments (or GJ-connected networks) in a normal brain. One of these compartments (or “networks”) is the neuronal compartment in which neurons communicate between themselves through GJs (electrical synapses), mostly made of Cx36 [25,26,27]. A second one, the vascular compartment is constituted by capillary endothelial cells (endothelium), which are connected between themselves and with pericytes via Cx37, Cx40, and Cx43. The 3rd GJ compartment is localized between the two previous ones and constitutes a panglial compartment (“panglial syncytium”). In this panglial syncytium, the cells expressing Cxs include astrocytes (Cx43, Cx30 and possibly Cx26) and oligodendrocytes (Cx32, Cx29, Cx47), resulting in the formation of GJs directly connecting astrocytes, oligodendrocytes and ependymocytes (Cx43) [41,46,63,144,145,146,147]. Other crucial cell types for brain function, including microglia and stem cells, do not seem to constitute such firmly established GJ compartments. However, since these cell types express Cxs and are able to make GJs, at least occasionally, it is possible to consider them as members of “putative” GJ compartments. As presented below, the spatial organization of “well-established” or “putative” GJ compartments have functional consequences (Figure 2).

##### The Neuronal Gap-Junction Compartment

The simplest GJ compartment in the brain is the neuronal compartment, which is only made of neurons connected by electrical synapses (Figure 2). This neuronal compartment is quite extended in the embryonic CNS where electrical synapses are numerous. Then, electrical synapses become limited during development [22,24,25,148,149,150,151,152] up to being restricted, in adults, in particular brain areas like the cerebral cortex (inhibitory GABAergic interneurons) [153,154,155,156,157], hippocampus (synchronous activity among inhibitory interneurons), reticular thalamic nucleus (synchronization of burst-firing), suprachiasmic nucleus (circadian behavior) [158,159], hypothalamus (pulsatile oxytocin release) and brainstem (control of respiratory modulation) [27]. 

All these electrical synapses are mostly made of Cx36, even if Cx31.1, Cx45, Cx57 and others have been found in murine neurons of some brain regions such as the retina [30,31,32,33,34,35]. Therefore, consistent with the role of neurons in which Cx36 is expressed, Cx36 GJs mostly promote the synchronization of interconnected neurons [118,160]. If such a synchronization is required, for example, for circadian rhythm, the presence of inhibitory networks coupled by Cx36 GJs permits resistance to the development of epileptiform synchronization. In relation to this, it is interesting to note that mutations in the regulatory promoter of the Cx36 gene (*GJD2*) are linked to juvenile myoclonic epilepsy (Table 2) [38,39]. In addition to their function in limiting excitability, neuronal GJs also play a major role in learning and memory. Indeed, Cx36-KO mice exhibit impaired short- and long-term memory as well as reduced motor learning (Table 2) [36,37]. Cx31.1, which is expressed in dopaminergic neurons of the substantia nigra pars compacta [161] and striatal output neurons [29], seems to play a role in exploration of novel environments and object recognition since these functions are either elevated or impaired, respectively, in Cx31.1 KO mice [23,162].

A general property of these electrical synapses is their high degree of plasticity, which makes them capable of modifying their coupling strength under various physiological conditions [27]. Cx36 has also been reported to act not only as a GJ protein but also as HC components releasing ATP during depolarization in cortical spreading depression [28]. Further studies could verify this.

##### The Panglial Syncytium

As a GJ compartment, the panglial syncytium is made of astrocytes, oligodendrocytes and ependymocytes. Because of their abundance and roles, only the first two cell types will be considered (Figure 2). 

Astrocytes are the most numerous cells in the panglial syncytium. They represent an intermediate layer between neurons and blood vessels since they project, for some of them, extensions to synapses (tripartite synapses) as well as to capillaries they surround by their endfeet [163]. Astrocytes are coupled extensively by GJs mostly made of Cx43 but also Cx30, which seems to be present more in grey matter [40] where there seem to be mainly homotypic/homomeric Cx43/Cx43 or Cx30/Cx30 [164]. Contrary to Cx30, whose expression is delayed during brain maturation, Cx43 is more homogeneously present in the astrocyte network of white matter, becoming the most abundant Cx in CNS [40,41,165,166,167,168]. Expression of Cx26 has also been reported in rodent astrocytes after birth, where it was observed in GJs between astrocytes and with oligodendrocytes [41]. However, this expression of Cx26 in astrocytes is still debated as deletion of both Cx43 and Cx30 in astrocytes causes pathological conditions [60], whereas lacZ under the Cx26 promoter is not expressed in astrocytes [43].

As the other important part of the panglial syncytium, oligodendrocytes form few GJs with astrocytes, mainly to those they depend on for homeostatic and nutrient support [169]. In oligodendrocytes, 3 Cx isoforms are expressed (Cx29, Cx32, Cx47) exhibiting different subcellular localizations. Cx47 colocalizes with Cx32 in GJs present in the soma connected to astrocytes (heterologous GJs) while Cx29 and Cx32 are concentrated in reflexive GJs connecting the concentric myelin layers. Cx47 is also present in small GJs linking myelin to astrocytes but not in deeper layers of myelin [62,65,66]. Apparently, oligodendrocytes also communicate, but to a much lesser extent, through homotypic Cx47 GJs, with other oligodendrocytes and their precursors in white matter [67]. Therefore, the only way most oligodendrocytes can communicate between each other is indirectly through GJs with astrocytic «intermediaries» [170,171].

This particular GJ network, in which at least 5 Cx isoforms are involved, plays fundamental roles for neuron survival and activity. Since neurons are distant from blood capillaries, their energy supply (glucose and lactate) cannot be transmitted directly to them but has to pass through astrocytes whose endfeet surround the basal membrane of the vessels [172]. Glucose molecules enter from blood to astrocytic endfeet by specific transporters and then traffic from astrocytes to astrocytes through Cx43/Cx30 GJs to be released by transporters to the immediate proximity of neurons, where uptake occurs. Apparently, in astrocytes, glucose is transformed through a glutamate-induced glycolytic degradation pathway into lactate, which is transferred to neurons via monocarboxylate transporters [45,173]. Moreover, glucose diffusion passing through astrocytes is not only directed to supply neurons, but is also distributed, through GJs, to oligodendrocytes and ependymocytes.

This energy supply in neurons is essential for the generation of action potentials. Due to neuronal activity, these action potentials increase extracellular K^+^ concentrations, which could be deleterious for neuronal function. Extracellular K^+^ ions are taken up by astrocytes and redistributed though astrocyte network following their gradient before being released in the interstitial space. Interestingly, GJIC (ionic coupling) of the astrocytic networks plays a particular role in this phenomenon by equalizing the astrocytic membrane potential (V_M_) to levels comparable to its neighbours [174]. This minimizes V_M_ depolarization due to elevated local [K^+^]_e_ and thereby maintains a sustained driving force for highly efficient K^+^ uptake. Thus, GJIC permits to maintain a constant extracellular K^+^ microenvironment by achieving isopotentiality in astrocytic networks [174]. Astrocyte endfeet contacting capillaries can also release excess K^+^ ions and associated osmotic water via Kir4.1 potassium channels [175] and AQP4 water channels that are concentrated in the endfoot plasma membrane [176,177]. Involved in the redistribution of extracellular ions and water, Cx43-mediated GJIC has been reported to regulate astroglial volume [48,60,178]. Because astrocytes occupy 50% of the brain volume [179], changes in astrocytic volume classically lead to opposite variations in extracellular space volume, which can affect neuronal activity by altering the concentration and diffusion of extracellular ions and neurotransmitters. Oligodendrocytes also participate in this K^+^ buffering through their myelin sheath all along the axon of neurons [70]. After entering the innermost cytoplasmic layer of myelin, K^+^ ions pass through successive paranodal loops through Cx32/Cx29-containing reflexive GJs to the outermost layer of myelin, then into the astrocytes through heterotypic GJs (Cx47/Cx32: oligodendrocyte side; Cx43/Cx30: astrocyte side). Therefore, through a complex and unique communicating network combining a succession of dense autologous, heterologous, and then homologous GJs, both astrocytes and oligodendrocytes participate to K^+^ siphoning, avoiding the excess of extracellular K^+^ ions, which do not reenter into neurons by Na^+^/K^+^ ATPase pump activity [63]. Failure to remove sufficient K^+^ results in changes in resting membrane potential allowing spontaneous action potentials.

Moreover, if reflexive GJs permit the diffusion of K^+^ ions through the myelin layers of oligodendrocytes, they also permit ionic homeostasis necessary for myelin integrity. Any lack of function due to mutation of Cx47 or Cx32 may cause myelin vacuolation or dismyelination [70]. This results in loss of velocity of action potentials along neuronal axons as it is the case in the Pelizaeus–Merzbacher-like disease [Cx47 gene (GJC2) mutation] [77,78] or Charcot–Mary-Tooth disease [Cx32 gene (GJB1) mutation] [75,76]. Astrocytes also contribute to this function by relaying to their network this ionic homeostasis. Any disturbance of astrocytic GJIC may then provoke dismyelination among oligodendrocytes [60]. Moreover, GJIC-deficient astrocytes display a reduced threshold for the generation of epileptiform events [47,60]. Therefore, the panglial syncytium entirely contributes to the efficient propagation of action potentials along the axons by maintaining myelin integrity.

Neurotransmitter buffering is necessary to maintain efficient synaptic transmission. Such a buffering is due to astrocytes present in the so-called «tripartite synapses». By adequate membrane transporters, astrocytes uptake neurotransmitters present in the synaptic space and enable them to diffuse through the astrocytic network before their eventual recycling to the neurons. This process has been documented for glutamate [45,173]. This excitatory amino acid is cleared from the neuronal synapses by astrocytes via glutamate transporters, and is converted into glutamine, which is released and in turn taken up by neurons. Metabotropic glutamate receptor activation on astrocytes triggers a variety of responses via increases in cytosolic Ca^2+^, such as Ca^2+^-dependent glutamate release from the astrocytes, which modulates the activity of both excitatory and inhibitory synapses. In vivo studies have identified the astrocytic endfoot processes enveloping the vessel walls as the center for astrocytic Ca^2+^ signaling and it is possible that Ca^2+^ signaling events in the cellular component of the BBB are instrumental in modulation of local blood flow as well as substrate transport. 

By facilitating K^+^ and neurotransmitter buffering, the maintenance of an efficient panglial syncytium is therefore necessary to keep the immediate environment of neurons compatible with their function and survival. 

##### The Vascular Gap-Junction Compartment

Although the human brain accounts for only 2% of total body weight, it demands 20% of the overall energy produced by the body. Since the brain lacks the capacity to store energy, a vast vascular compartment responds to the continuous demand of oxygen and glucose (and waste exit) necessary for neuronal survival and activity and for other cells of the CNS [180]. In an adult brain, it is estimated, by stereological approach, that such energy supply and waste exit are carried out by a network of 600 km of capillaries (7 µm diameter) representing an exchange surface of approximately 15–25 m^2^ [180,181,182,183]. Such a high density of capillaries places each neuron within 20 μm from a capillary. As seen above, neurons require a tightly regulated extracellular environment to function appropriately, and the 20 μm proximity between neurons and capillaries makes them susceptible to possible chemical fluctuations (ions, hormones, neurotransmitters, amino acids, etc.). Causes of such fluctuations are numerous and may even include circulating neurotoxic substances from external origin (drugs, pollutants, etc.). As a protection, the exchange of substances between blood and brain is tightly controlled by the BBB, which, thus, plays fundamental roles such as regulating ion concentrations, facilitating nutrient transport, and preventing toxic substances [184].

Anatomically, BBB is therefore constituted by a 600 km syncytium of vascular endothelial cells (endothelium) lining the lumen of the capillaries (Figure 2). A thin layer of basal membrane separates endothelial cells from less numbered pericytes (ratio 1:3). Despite this thin basal membrane, endothelial cells and pericytes are nevertheless in contact from place to place through GJs [185,186]. Such GJIC and also paracrine communication are involved in pericyte contraction, which contributes to the local regulation of blood flow by controlling capillary diameter [83,86]. Endothelial cells and pericytes are enveloped by the basal membrane of the capillary, which is almost entirely enclosed by astrocyte endfeet (Figure 2). Importantly, the perivascular space delimited by the astrocytic endfeet is part of the so-called glymphatic system involved in draining interstitial fluid and removing metabolic wastes [187,188,189].

Substances can cross the BBB only via paracellular and transcellular pathways restricted by the presence of adherens and tight junctions between endothelial cells. The transcellular trafficking of endocytic vesicles carrying substances from the apical membrane through the cytoplasm before exiting the basal membrane is more limited than in the rest of the organism [180,190]. Endothelial cells extensively communicate between each other and with pericytes through GJs made of Cx37, Cx40 and Cx43 (Figure 2) [80]. This particular GJ compartment is implicated in BBB function since the presence of functional Cxs is necessary for its maintenance [81]. Moreover, expression of Cx30 and Cx43 at the astrocytic endfeet during development coincides with postnatal maturation of BBB. The absence of these Cxs in the astrocyte endfeet weakens the BBB when hydrostatic vascular pressure is increased [49]. Similarly, the presence of Cx43 in astrocytes helps keep the endothelial barrier closed for immune cell infiltration [191]. However, Cx43 on the endothelial side would have an opposite effect on BBB permeability as observed in a murine model of the familial cerebral cavernous malformations type III (fCCM3). This disease is caused by loss-of-function mutations in *CCM3* that result in dilated capillary beds, which are susceptible to hemorrhage because of BBB disruption. Interestingly, Cx43 appears to be up-regulated in developing CCM3 lesions and through its interactions with ZO-1 would favor BBB breakdown by limiting tight junction formation [192]. Similarly, the impairment of pericyte-endothelial interaction increases BBB permeability [193].

The situation is probably more complex because GJ channels and Cx HCs of BBB appear to play opposite roles in the regulation of its permeability. GJIC contributes to maintaining BBB integrity, while HCs are associated with ATP signaling release and necessary to generate Ca^2+^ oscillations linked to BBB disruption induced by proinflammatory signals [194]. Moreover, it is quite possible that Cx HCs then affect the BBB transcellular pathway by modifying [Ca^2+^]_i_, which is involved in the vesicular pathway [194]. Such a situation would depend then on the neuroinflammation status since the release of gliotransmitters or cytokines by activated astroglial and microglial cells affects GJIC and activates Cx HCs [195]. In this situation, it would be interesting to determine whether Panxs, which share many common functions with Cx HCs, might also be involved. This is important because disruption of the BBB is associated with many diseases of the CNS associated with neuroinflammation such as neurodegenerative diseases like AD [196,197,198,199,200]. BBB disruption has been also observed in PD [198,201,202,203], epilepsy, seizures [204,205,206], and brain tumors [207,208,209]. Moreover, it is also possible that BBB disruption affects the glymphatic-draining function by disturbing the perivascular space. Disturbance of such a process seems to be involved in AD by preventing the efficient clearance of amyloid-β deposits [210,211]. 

##### Other Putative Gap-Junction Compartments in Brain

The GJ compartments that are functionally described above are well established after birth and for the entire life. The integrity of such an organization is maintained and protected against any cell damage. This protection is assured by cells playing complementary roles by eliminating pathogens and damaged cells (microglia) and by replacing damaged cells (stem and precursor cells). Either scattered inside brain parenchyma (microglia) or concentrated in particular niches (stem cells), these cells do not seem to constitute a well-established and permanent GJ compartment. However, they express Cxs and are therefore able to communicate either directly through GJs or through paracrine communication with other cell types, depending on their activation.

- Stem Cell Niches

**Figure 3:**
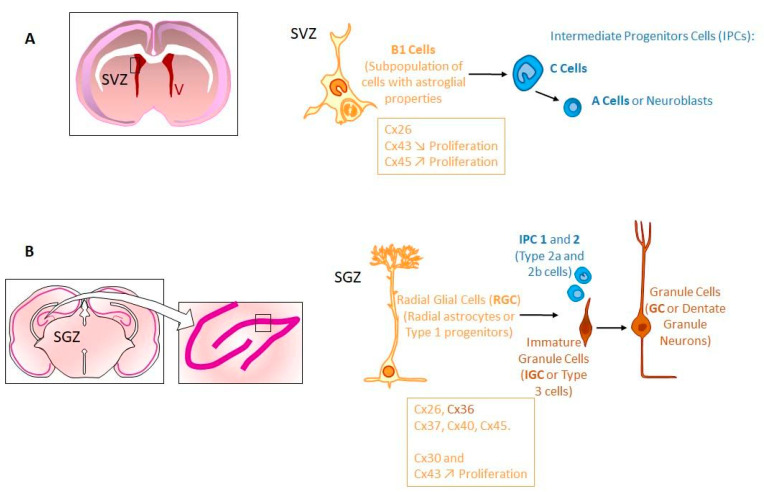
Stem cell niche and neurogenesis in adult. Neural stem cells (NSCs) are mostly retained in two regions: Sub-ventricular zone (SVZ) and sub-granular zone (SGZ). (**A**) Frontal cross-section of the adult brain showing the location of the SVZ, in walls of the lateral ventricles (V). In the SVZ, NSCs correspond to type B1 cells. B1 cells express Cx26, Cx43 and Cx45. While Cx43 expression negatively regulates cell proliferation, Cx45 exhibits an opposite role. These B1 cells generate intermediate progenitor cells (IPCs) corresponding to C cells that divide to generate neuroblasts (type A cells). (**B**) Frontal cross-section of the adult brain showing the hippocampal formation. The insert indicates the location of the dentate gyrus. The adult dentate gyrus contains radial glial cells, which are polarized cells with their cell body in the SGZ. Radial glial cells (RGC or RC, type 1 cells) generate IPCs (IPC1 and IPC2 or type 2a and type 2b cells), which differentiate into immature granule cells (IGCs or type 3 cells) and mature granule cells (GC). In SGZ, Cx26, Cx30, Cx36, Cx37, Cx40, Cx43, Cx45 are expressed in various NPCs. Interestingly, Cx30 and Cx43 seem to be active regulators of hippocampal NPC proliferation while Cx43 itself is a negative regulator of proliferation in SVZ [258].

After birth, neurogenesis can occur in limited niches of the mammalian brain (Figure 3). In adults, those niches are located in the subventricular zone (SVZ), the subgranular zone (SGZ) of the dendrite gyrus of the hippocampus [24,101,104], and in the hypothalamus (dorsal α1, α2 regions and in the «hypothalamic proliferative region», adjacent to the median eminence, in the β region) [212,213]. The restriction of the neurogenesis areas is the result of a progressive developmental process well described for the SVZ [141,142,214,215,216,217,218,219].

Once formed, the SVZ lines the walls of the lateral ventricles (LVs). It contains neural stem cells (type B cells) [220] located under the ependymal layer of the ventricle [221]. Those cells have projections towards the CSF in the LV, and also contacts with blood vessels (BVs) of the SVZ plexus [217]. This situation allows type B cells receiving signals from CSF and blood to form transit-amplifying NPCs (type C cells) in asymmetric divisions, which divide to form neuroblasts (type A cells) [220,222,223]. In humans, during the first postnatal months, those neuroblasts migrate to the frontal lobe tangentially close to the walls of LVs and along BVs, through a mechanism probably controlled by astrocyte-released soluble factors [224,225,226]. Then, these neuroblasts individually reach cortical tissue where they differentiate and contribute to inhibitory circuits. These late-arriving inhibitory interneurons could contribute to developmental plasticity since the disruption of their postnatal migration may underlie neurodevelopmental disorders [227,228,229]. Interestingly, such a migration coincides with the such a migration happens during the first months after birth, a critical period for human brain development, when children begin to interact with their environment. This is especially true for the human frontal lobe, which is so important for such interactions and social behavior. Afterwards, neurogenesis and migration decline, disappearing by two years of age [228,229,230,231,232]. This cessation of postnatal neurogenesis in the forebrain seems to be particular to human [233,234]. In addition, SVZ type B cells can also generate oligodendrocyte precursors that contribute to the maintenance of the oligodendrocyte population in the neighboring corpus callosum, striatum and fimbria-fornix [235,236].

Even if it is not well understood yet, cell-cell interactions seem to play an important role during the maturation and maintenance of stem cell niches. Indeed, postnatal NPCs and immature neurons of the SVZ express several types of Cxs (Cx26, Cx43, Cx45) [107,108,237,238,239,240,241,242,243,244]. GJIC has been demonstrated not only between NPCs (radial glial cells) but also between them and astrocytes or microglia [237,239,240,241,245]. Apparently, Cx43 expression increases with postnatal age in SVZ NPCs negatively regulating cell proliferation, contrary to its promoting role during embryogenesis [242,243,246,247]. In contrast, in those cells, Cx45 exhibits an opposite role in NPCs by inducing cell cycle reentry via ATP signaling [243]. Moreover, it has been demonstrated that GJIC mediated by those Cxs is involved in the migration of NPCs within the SVZ [238]. In the postnatal hippocampus, almost the same Cxs are expressed (Cx26, Cx30, Cx37, Cx40, Cx43, Cx45) in various NPCs. However, the expression changes during differentiation (Cx36 appearing in immature neurons). Interestingly, Cx30 and Cx43 seem to be active regulators of hippocampal NPC proliferation while Cx43 itself is a negative regulator of proliferation in SVZ [150,248,249,250,251]. Although the majority of Cxs are docked to function as GJs, unapposed HCs have also been documented on the surface of different cell types [252,253].

By the presence of stem cell niches, the postnatal brain exhibits more plasticity than previously thought and this has implications for memory, learning, and the pathogenesis of neurodegenerative diseases [254,255,256]. For instance, in humans, hippocampal neurogenesis persists during very late decades of life while it is significantly declined in AD patients [257]. The fact that NPCs persist in the adult mammalian brain and can integrate into brain circuity is proof of adult structural plasticity at the cellular level [5]. Interestingly, adult neurogenesis can be regulated by several behavioral factors like running, for instance, which, contrary to stress, induces such a phenomenon. Other behaviors, like learning, seem to have more complex effects, suppressing neurogenesis at some stages (proliferation of progenitor cells), and increasing it at others (differentiation and survival) [5]. 

In conclusion, any disruption of proliferation and differentiation of neural stem cells and migration of their progeny may contribute to neurodevelopmental and neurocognitive deficits such as autism [227,228,229]. If GJIC is important for such proliferation, differentiation, and survival of neural progenitor cells, any inhibition could indeed have deleterious effects.

ii. Microglia

While they belong to the glial cell types (5–20% of glial cells) with macroglia (astrocytes and oligodendrocytes), microglia are not part of the panglial syncytium from which they differ in their embryonic origin [259,260]. So far, despite the fact they do express Cxs, there is no evidence that microglia establish permanent GJs with astrocytes and/or oligodendrocytes. Therefore, microglia can be considered as distinct from macroglia. 

As the resident immune cells in the CNS, microglial cells play important roles in health and disease. In the healthy CNS, they exhibit a ramified morphology with multiple fine processes that survey their surrounding microenvironment. If a pathological stimulus appears, as a result of any type of injury or disease, microglial cells acquire an activated phenotype in which their morphology changes towards a hypertrophic or ameboid-like appearance and their functional behavior is altered [261,262,263,264]. Along with these features, mounting evidence suggests that microglia continually extend and retract their cell processes toward and from synapses, being part of a new spectrum of unexplored capabilities, such as synaptic pruning, maturation and remodeling, as well as modulation of synaptic transmission and plasticity [265,266,267]. Of note, when severe or chronic brain injury occurs, microglia become activated, triggering a widespread release of inflammatory molecules, facilitating the engagement of non-resident brain cells implicated in the innate and adaptive immune function [268].

The main function attributed to activated microglia is the phagocytosis of pathogens, degenerating cells or debris. However, they perform other functions in the activated state, such as removal of dysfunctional synapses (synaptic stripping) [269] or regulation of synaptic plasticity [270]. As part of their activation, microglial cells secrete reactive oxygen species, cytokines and growth factors that influence the pathological process and the subsequent tissue regeneration [261,262,263,271]. Therefore, it is well accepted that surveillant microglia, in addition to their monitoring role in the healthy CNS, can also influence neuronal structure and function contributing to the maintenance of neural circuits [272,273].

Microglial cells are widespread in brain parenchyma but also present, at high density, in stem cells niches. Expressing Cxs (Cx32, Cx36, and when activated, Cx43) [88,89,90,274,275], they can potentially communicate through GJIC with other cell types such as neurons [88] or NPCs in co-cultures [237,276]. In vitro, they form GJs in response to inflammatory stimuli such as cytokines or bacterial pathogens [90,91]. However, a study performed in vivo could not demonstrate the presence of GJIC between microglial cells or between microglia and neurons [277]. Among the microglial Cxs, Cx43 seems to be responsible for the formation of functional GJs [90,278]. Despite the fact that biological significance of microglial coupling remains elusive, it has been hypothesized that GJs are crucial for ruling dynamic changes in microglial phenotype, the exchange of antigen peptides between activated microglia or the cross-presentation of antigens to T cells [98]. Some studies showed that TNF-α-mediated upregulation of Cx HCs contributes to the exacerbated release of glutamate and subsequent neuronal beading and death [279]. Moreover, different inflammatory agents [Aβ, lipopolysaccharide (LPS) and ATP] have been described to increase the opening of HCs formed by Cx43 and Cx32 [280].

In the particular case of stem cell niches, the ability of NPCs to form functional GJs with microglia can be physiologically and pathologically relevant. In healthy conditions, close proximity between those cell types has been documented in the SVZ niche [93,94,95]. Indeed, microglia, which are denser in SVZ, establish a bilateral cross-talk with NPCs that affects both microglial activation state and NPC proliferation and differentiation [93,94,95,96,97]. The inflammatory response triggered by host microglial cells is known to preserve implanted NPCs in an undifferentiated state [281]. Interestingly, microglia in the SVZ display a unique phenotype characterized by specific morphology, low expression of purinergic receptors and microglial markers such as Iba1 and CD68 [282,283] and the release of a distinct set of cytokines [282,284]. Therefore, microglia in the SVZ may influence NPCs behavior through particular characteristics, which may confer on them specific properties and putative roles in the control of SVZ neurogenesis. In line with this assumption, soluble factors released from microglial cells direct the migration of SVZ NPCs and increase the proportion of new neurons in SVZ embryonic and adult cultures [97,285,286,287]. It is interesting to note that the effect of microglia in the promotion of neurogenesis is produced in the early postnatal period, when microglia display mainly an amoeboid morphology and reach their maximum levels before decreasing to adult numbers and adopting a resting ramified morphology [97]. Moreover, it has been shown that microglia are also involved in hippocampal neurogenesis through the phacocytosis of newborn cells that do not integrate the existing circuits and become apoptotic [288]. To our knowledge, it has not been confirmed whether GJIC is involved in this phenomenon.

#### 2.2.2. Connexin Paracrine/Autocrine Functions in Brain

During recent years, the involvement of Cxs in brain physiology has been shown to not be limited to GJ formation. Indeed, Cxs can play important physiological (and pathological) roles through their HC activity, which is responsible for paracrine/autocrine communication. Interestingly, this Cx-mediated paracrine communication seems to be involved in communication pathways between the GJ compartments defined above. 

Such physiological Cx-HC activity (opening) has been mostly described in the panglial syncytium and particularly among astrocytes. So far, at least two physiological phenomena have been described in wich Cx HCs are involved. One is in relation with the activity of the neuronal compartment (intercompartment paracrine communication) and the other takes place with microglia, which is not a GJ compartment, in the context of neuroinflammation.

##### Connexin Paracrine/Autocrine Communication and Neuronal Activity 

Both decreased [Ca^2+^]_e_ [52,53] and increased K^+^ may trigger the opening of astrocytic Cx43 HCs [54]. In the case of [Ca^2+^]_e_, a transient drop in the range of 0.4–0.6 mM is sufficient to induce such an opening. Once open, Cx43 HCs permit ATP release [289], which increases cytosolic Ca^2+^ in the same astrocytes (autocrine effect) or in the surrounding astrocytes (paracrine effect) through the activation of metabotropic purinergic P2Y_1_ receptors [290]. In parallel, this paracrine propagation is intensified among the astrocytic network by direct intercellular propagation of IP_3_ and Ca^2+^ waves passing across Cx30/Cx43 GJs [48,55]. Therefore, mobilization of intracellular Ca^2+^ stores represents the primary pathway by which astrocytes respond to neural activity. Finally, intercellular Ca^2+^ wave propagation progresses through intra- and extracellular signaling pathways, which mutually influence each other in synergistic ways. In turn, increased cytosolic Ca^2+^ is essential for the release of gliotransmitters. A consequence of the opening of Cx43 HCs is the extracellular release of glutamate, which activates postsynaptic neurons through NMDA channels. It seems that Cx43 modulates presynaptic glutamate vesicular pools by mechanisms which are not completely elucidated [56]. Astrocytic HCs are involved therefore in the modulation of neuronal activity, and more precisely of synaptic strength and plasticity [44]. 

Thus, such neuron/astrocyte pathways acting through Cx HCs have probably important physiological consequences on brain function, which are not completely analyzed yet. Some studies appear to reveal only the emerging part of the iceberg as, for instance, the release of gliotransmitters through astrocytic Cx43 HCs, which seems to be necessary for fear memory consolidation in the basolateral amygdala [291]. Thus, purinergic signaling molecules released by Cx HCs acting on neuronal and glial P2X and P2Y receptors comprise a fundamental component of synaptic plasticity [21]. On the pathological side, comparable decreases in [Ca^2+^]_e_, necessary to activate astrocytic HC pathway, have been noted during epileptic activity and also in ischemia [290,292]. 

##### Connexin Paracrine/Autocrine Communication and Microglia Activation 

Cx HCs can be activated by IL-1β or TNF-α pro-inflammatory cytokines, which are secreted by microglia during the neuroinflammation process [50,293]. Neuroinflammation is a response of the brain to a pathological situation. This process is associated with reactive gliosis characterized by microglial activation [262] and astrocyte reactivity [294]. For example, the endotoxin LPS, which is present in the external membrane of Gram-negative bacteria, is recognized by TLR4 of microglia and activates the secretion of pro-inflammatory cytokines (IL-1β and TNF-α) that activate [295], in turn, astrocytic Cx43 HC [50,51]. The opening of astrocytic Cx43 HCs results in increased [Ca^2+^]_i_ and extracellular glutamate concentrations associated with a decrease (50%) in excitatory synaptic transmission in the CA1 area of the hippocampus [51]. This illustrates that activated microglia may modify neuronal activity [51,87,90,296,297,298]. Microglia respond to inflammatory cytokines by upregulation of glutaminase and glutamate release due to HC activation [87], which induces downregulation of the astrocytic glutamate aspartate transporter, GLAST [298]. Therefore, microglial HCs alter extracellular glutamate concentration and astrocyte-dependent glutamate buffering in inflammation. Inflammation-induced dysregulation of GJs not only impacts glutamatergic neurotransmission [51,87,297,298], but also underlies disorders of excitation, such as epilepsy [145]. Therefore, activation of microglia may induce deleterious effects. This is shown in the case of *Staphylococcus aureus* infection, which leads to alteration in GJIC and HC activity of astrocytes [299], and in the case of AD, in which amyloid-β peptide activates microglial cells and leads to neuronal death through a mechanism involving astrocytic Cx43 HCs [92].

It would also be important to check carefully whether what is attributed to Cx HCs is not performed by Panx-made channels.

### 2.3. Possible Consequences of Connexin Dysfunction in Brain 

As shown above, there is increased evidence indicating that Cxs are strongly involved in brain physiology either as GJs or as HCs. With the exception of microglia, GJIC defines three permanent functional compartments able to communicate between themselves via HC paracrine communication. Therefore, in the neuronal compartment, for instance, Cx36 GJs of the GABAergic interneurons allow for rapid and expansive inhibition via electrically coupled inhibitory syncytia that are calibrated to depolarizing activity [118,156,300]. More generally, several studies indicate that Cxs are critical to maintaining physiologic neuronal excitability, resistance to seizure, and may be central to hippocampus and amygdala based learning [144,156,291]. 

In the panglial syncytium, the presence of GJs supports physiological resting membrane potential through spatial buffering of K^+^ and constrains epileptiform bursting [46,63,144,145,146,147]. Astrocytes are central to the ability of the panglial syncytium to carry out this function and recent evidence illustrates that dysregulation of astrocyte Cx43 GJs may drive the pathogenesis of some human temporal lobe epilepsy. Without efficient GJs, astrocytes could not optimize the interstitial space for synaptic transmission by tight control of water and ionic homeostasis. Astrocytes are active participants in the tripartite synapse and modulate synaptic activity in hippocampus, cortex, and hypothalamus. Therefore, astrocytes include both supportive functions as well as active modulation of neuronal output activity [301]. It is clear that multitask functions of astrocytes are essential for higher brain function and Cxs play an essential role in all these functions. Lack of GJIC could lead to movement of water from the vasculature into the parenchyma across an intact BBB, leading to astrocyte swelling and decreased extracellular space. Lack of GJIC could also lead to unstable V_M_ that would perturbate K^+^ uptake [174]. The astrocytic GJ network then appears to play an important role in both ionic and water regulation in the brain. On the other side, purinergic and glutamatergic gliotransmission, which can modify neuronal activity, relies heavily on autocrine and paracrine signaling by Cx HCs [21].

The fundamental roles of the other important cellular components of the panglial syncytium, the oligodendrocytes (K^+^ buffering and myelin integrity), are also a consequence of the presence of functional Cxs. For instance, mutations in oligodendrocyte-expressed Cx genes result in phenotypes indistinguishable from inherited hypomyelinating leukodystrophies, which are characterized by impairment of myelin sheath formation, inflammation, and sensorimotor neurological deficits [146,302,303]. A mutation in the Cx47-gene promoter results in Pelizaeus–Merzbacher-like disease [78], while altered Cx32 expression leads to symptomology closely resembling Charcot–Marie-Tooth disease [76]. Moreover, mice lacking Cx32 and Cx47 exhibit more pronounced myelin pathology, which is accompanied by loss of oligodendrocytes and action tremors progressing into tonic-clonic seizures and mortality by the 6th postnatal week [66,70]. Disruption of GJs between oligodendrocytes and astrocytes leads to myelin defect as shown in Cx47—KO mice in which myelin vacuolation is observed in the optic nerve [66]. 

Loss and activation of astrocytes, oligodendrocyte loss, myelin vacuolation, inflammation, and invasion of phagocytic cells are also common to GJIC disorders [74,146,303], suggesting a role for an intact panglial syncytium in supporting survival of its members and homeostatic functions. However, while non-channel properties of Cxs have been explored in the context of neuronal differentiation [246,251] and glutamatergic transmission [56], the mechanism whereby Cxs support glial survival remains poorly understood.

Since Cxs are implicated in various functional aspects of brain cells, it is not surprising that many cerebral pathologies (hereditary diseases, injury, inflammation, epilepsy, neurodegenerative pathologies, benign and malignant tumors) have been linked to GJIC and/or Cx dysfunctions. Knowing that Cx function is very sensitive to various stimuli, a fundamental question is whether chronic exposure to chemical pollutants may be involved in the observed increased incidence of some of these pathologies by altering Cx functions.

## 3. Inhibition of Gap-Junctional Intercellular Communication by Environmental Chemical Pollutants

GJ channels, and in particular those composed of Cx43, the predominant Cx in astrocytes, are emerging as major targets of an increasing number of environmental chemical pollutants. Among such pollutants are pesticides, bisphenol A (BPA), phthalates, polychlorinated biphenyls (PCBs), polybrominated biphenyls (PBBs), dicumylperoxide (DCP), perfluorodecanoic acid (PFDA), perfluorooctanoic acid (PFOA), perfluorooctane sulfonic acid (PFOS), 1-monolaurin, polycyclic aromatic hydrocarbons (PAH), 2, 3, 7, 8-tetrachlorodibenzo-p-dioxin (TCDD), fine particulate matter with aerodynamic diameters less than 2.5 µm (PM_2.5_) and toxic heavy metals (Table 3). The capacity to inhibit GJIC of some of these chemical compounds, as well as others, was known for long and reviewed decades ago [304,305]. 

Because of their lipophilic properties, some of these environmental chemical pollutants are able to cross the BBB and reach the CNS [306,307]. Although such pollutants are able to modulate GJIC, several underlying direct or indirect mechanism(s) have been proposed. For some of them, an insertion into the membrane lipidic bilayer, with resulting modification in the microenvironment of the membrane channels and a non-specific change in the membrane biophysical properties can be proposed [308]. Another potential mechanism of action is an inhibition of GJIC by physically blocking the GJ channel pore [309,310,311]. Changes in the Cx gene expression, endocytosis and degradation were also observed in response to several pollutants [312,313,314,315,316,317,318,319,320,321]. The last potential mechanisms of action are through non-classical estrogen receptors (ERs) [322,323] or through a toxic action on the aryl hydrocarbon receptor (AhR) [312]. Moreover, several intracellular signaling pathways were found to be involved in GJIC dysregulation including phosphatidylcholine-specific phospholipase C (PC-PLC) and the MAPK-kinase MEK [324,325,326,327,328,329,330]. 

### 3.1. Pesticides

Pesticides comprise substances from many different chemical groups with potent biological activity and a large number of different mechanisms of action. Pesticides include herbicides, which are the most common, insecticides, antiparasitics and fungicides. Many of them act as endocrine disruptors and are GJIC modulators.

#### 3.1.1. Insecticides

##### Organochlorine Insecticides

DDT (1,1,1-trichloro-2,2-bis(4-chlorophenyl) ethane) is a persistent organochlorine chemical that can still be widely found in soils, in aquatic environments, in sediment and in various animal species decades after its use has been discontinued. DDT was the most used organochlorine insecticide in the world since the 1940s for agricultural purposes and for vector-borne disease control such as malaria and typhus, until its banishment in most countries, except in some developing countries in Africa and Asia. After application to soils, DDT may be lost through both volatilization and biodegradation. In the environment, dichlorodiphenyldichloroethylene (DDE) and dichlorodiphenyldichloroethane (DDD) are the major degradation products of DDT. Human exposure to DDT can occur by several routes, including ingestion of contaminated foods, the main source, skin absorption or through respiration and accidental contamination [352]. DDT and its derivatives are recognized as endocrine-disrupting chemicals [353]. Various studies have also shown that DDT and its metabolites (DDE and DDD) can inhibit GJIC in certain types of mammalian cell cultures (Table 3) [305,312,313,331]. In normal human breast epithelial cells, DDT seems to alter GJIC at the post-translational level. At non-cytotoxic concentrations of DTT that inhibit GJIC, a reduction in the number of GJ plaques and a reduction in the Cx43 phosphorylation level is observed. The same results are obtained after treatment with dieldrin, toxaphene, and PCB and PBB congeners. Moreover, there is no change in the steady-state levels of Cx43 mRNA after treatment with these chemicals. Interestingly, a “cocktail effect” is revealed in this study since specific mixtures of two of these chemicals showed that, while each alone do not inhibit GJIC at a given concentration, the mixtures do [313]. DDT inhibits GJIC between rat liver epithelial cells through a phosphatidylcholine-phospholipase C (PC-PLC) pathway independent of MEK [324]. 

Dieldrin is an organochlorine pesticide developed as an alternative to DDT and used extensively in the USA from the late 1940s until the 1970s, before being removed from the market in 1987. Unfortunately, dieldrin has a long environmental persistence and continues to be a common contaminant in soil. Dielbrin is an endocrine disruptor that displays only very weak binding to ER-α. Low dieldrin concentrations have the ability to inhibit GJIC in certain types of human and other mammalian cell cultures (Table 3) [305,332].

Chlordane (Octachloro-4, 7-methanohydroindane) was introduced in 1945 as the first chlorinated cyclodiene insecticide and became widely used as a soil insecticide against many agricultural pests and was also a widely used termiticide until it was banned in 1988 in the USA. Chlordane is persistent in soils and can volatilize. Chlordane, and other organochlorine pesticides like endosulfan, heptachlor and aldrin, markedly inhibit GJIC between cultured mammalian liver cells (Table 3) [305,333,334]. In the Chinese hamster cell line (V79), derived originally from lung tissue, chlordane does not inhibit GJIC as significantly as DDT or lindane [312].

Methoxychlor (1,1,1-Trichloro-2,2-bis(4-methoxyphenyl)ethane) is an organochlorine pesticide with known estrogenic activities in vitro and in vivo [354]. Methoxychlor inhibits GJIC in oviductal cells in monolayer culture after 1–5 h exposure to noncytotoxic concentrations of 16–64 µM [335]. Incubation of the rat liver epithelial cell line WB-F344 with 25 µM methoxychlor for 30 min almost causes complete inhibition of GJIC without affecting Cx43 phosphorylation status and its intracellular localization (Table 3). Moreover, this rapid inhibition of GJIC is mediated through MAPK ERK1/2, p38 and PC-PLC independently of ER or androgen receptor signaling [325].

Lindane (*γ*-hexachlorocyclohexane (γ-HCH)) was commercialized as an agricultural insecticide and is now largely banned due to its toxicity. In the environment, lindane was found in air, water, soil and dust, and the general population is also contaminated through diet [355]. This organochlorine has been reported to rapidly inhibit GJIC in different cell lines and under chronic exposure, induces a loss of GJ plaques and Cx43 endocytosis (Table 3) [312,314,315,316,317]. Moreover, the addition of lindane to open Cx43 HCs appears to have no effect on conductance [356]. It was further demonstrated that lindane regulates GJIC through a MEK1/2-dependent mechanism that is independent of PC-PLC [324]. 

Chlordecone (decachlorooctahydro- 1, 2, 4 -metheno-2H-cyclobuta (cd) pentalene-2 -one, kepone) is an estrogen-like organochlorinated pesticide with low agonist affinity for ER-α. Chlordecone is a known endocrine disruptor that is potentially carcinogenic and exhibits in vivo neurotoxicity, and perturbation of the serotonergic system. Following the chemical disaster in Hopewell, Virginia, in 1975, workers of the kepone manufacturer developed severe neurological disorders after exposure to high doses of the pesticide (motor disorders, mood, speech, immediate memory) [357]. In the French Antilles, it was intensively used from 1973 until 1993 as a substitute for lindane to control the banana root borer. Its persistence in the environment has resulted in a widespread contamination of Guadeloupean soils, water sources, animals and foodstuff leading to 95% of the population of Guadeloupe and 92% of that of Martinique being contaminated. Epidemiological studies showed that chronic low exposure to chlordecone is correlated with neurotoxicity and impaired neurobehavioral development (IQ point loss) in young children, characterized by a decrease in fine motor skills statistically significant only in boys [358,359,360]. However, there are no quantitative data for neurotoxic effects in adulthood in the French Antilles. Non-cytotoxic concentrations (4 µg/mL of kepone and 12 µg/mL of mirex for 3 days) of chlordecone and of its fully chlorinated structural analog, mirex (dodecachlorooctahydro1,3,4- metheno-2H-cyclobuta(cd)-pentalene), inhibit in vitro GJIC in Chinese hamster V79 cells, derived originally from lung tissue (Table 3) [337]. Moreover, in human embryonic palatal mesenchymal cells, chlordecone inhibits GJIC [338].

##### Toxaphene

Toxaphene is a complex mixture consisting primarily of chlorinated bornanes with lesser amounts of chlorinated camphenes, dihydrocamphenes, bornenes and bornadienes. Toxaphene was widely applied as an insecticide on cotton, soybeans, and corn, with more minor use as an acaricide. Although this compound is no longer produced commercially, it is still frequently found in the environment [361]. In normal human breast epithelial cells, toxaphene (10 µg/mL for 90 min) significantly inhibits GJIC (Table 3) and induces a reduction in the number of GJ plaques and in the Cx43 phosphorylation level [313].

#### 3.1.2. Herbicides 

Ioxynil (4-hydroxy-3,5-diiodobenzonitrile) is a benzonitrile herbicide that has been used actively worldwide to control weeds in agriculture since the 1970s. Studies addressing the fate of benzonitrile herbicides in the environment show that some metabolites of these herbicides are very persistent. It was reported to have endocrine disrupting properties [362]. In rat liver epithelial cell line IAR20, which express Cx43, phenolic and octanoic ioxynil are potent inhibitors of Cx43-GJs (Table 3) and induce degradation of Cx43 [318]. Another herbicide, alachlor, inhibits GJIC in WB-F344 rat liver epithelial cells in an MEK1/2 and PC-PLC-independent pathway [324]. 

#### 3.1.3. Fungicides

The fungicide vinclozolin (3-(3,5-dichlorophenyl)-5-ethenyl-5-methyl-2,4-oxazolidinedione) has been documented for its environmental endocrine disrupting activity [363]. Incubation of rat liver epithelial cells with 250 µM vinclozolin for 30 min causes almost complete inhibition of GJIC (Table 3). Moreover, vinclozolin induces rapid hyperphosphorylation and internalization of Cx43. The rapid inhibition of GJIC by the fungicide is mediated through MAPK ERK1/2, p38 and PC-PLC independently of ER or androgen receptor signaling [325]. Another fungicide, pentachlorophenol (PCP), inhibits GJIC in those cells in a MEK1/2 and PC-PLC-independent pathway [324]. 

### 3.2. Bisphenol A 

BPA [2,2-bis(4-hydroxy-phenyl)propane] is a ubiquitous synthetic estrogen without a steroid structure widely employed in a variety of consumer products in industrialized countries. Therefore, most people have been exposed almost continuously to sources of BPA through predominantly diet, inhalation, or dermal absorption. BPA has been found in the urine of almost all adults and children tested, in the saliva and serum of pregnant women, in foetuses, placenta, amniotic and follicular fluids and breast milk [322]. The unconjugated BPA level in human blood is 1.3–19.3 nM, and the human foetus exposure level is 4.4–13.2 nM [364]. Moreover, because of its lipophilic properties BPA has been demonstrated to undergo transfer to the CNS via the BBB [306]. While adult animals tolerate acute and high doses of BPA (e.g., >2–50 mg/kg b.w./day at multiple doses), harmful effects were further described in rodents exposed at a BPA concentration below the lowest observed adverse effect level also called “low-dose” (LOAEL, of 50 μg/kg/day for in vivo studies or 50 ng/mL) [310]. Then, in 2014, the European Food Safety Authority reduced this daily dose to 4 μg/kg, lower than that from dietary exposure. Although BPA has been found to have a lower affinity for classical nuclear ERs relative to 17-β estradiol (E2), it can also bind to non-nuclear ERs with estrogenic potency equal to E2 [365]. BPA can also act at low, environmentally relevant, doses, with estrogen-independent effects that can be transmitted by seven-transmembrane ER GPR30 or by estrogen-related receptor γ [322,323].

In several in vivo and in vitro systems, studies have demonstrated that BPA targets GJIC and interferes with Cxs expression, in particular Cx43 (Table 3). Western blot and immunofluorescence analyses showed that BPA exposure (45 µM, 24 h) reduced the level of Cx43 and delocalized it from the plasma membrane to the cytoplasmic compartment in SerW3 Sertoli cells [366]. An in vivo study showed a significant reduction in the expression of Cx43 in the testes of rats exposed neonatally to doses of BPA that were capable of impairing male fertility (0.6 to 10 µg/rat, or 100–1600 µg/kg body weight) [320]. When immature 20-days-old rats were treated by gavage for 5 or 6 consecutive days with a regimen that mimicked acute human short-term or accidental exposure to BPA (~2.5–4 μg/person/day) the integrity of the blood–testis barrier was reversibly perturbed. This observation was confirmed using primary Sertoli cells cultured in vitro and the disruption of Sertoli cell tight junction barrier by BPA was associated with a decline in the level of Cx43 [367]. In mouse immature oocytes, the entry of external Ca^2+^, probably through GJs, contributes to spontaneous Ca^2+^ oscillations, which are a marker of competence for fertilization. E2 and BPA at a 10,000-fold higher concentration (100 µM for a short-term exposure of 1 h) exert similar inhibitory action on spontaneous Ca^2+^ oscillations through unresolved mechanisms that could involve GJs [368]. The in vitro short-term exposure of rat cumulus cell-oocyte complexes (COCs) to BPA (20 ng/mL for 25 h) results in an overall increase in GJ transfer rate, which is coincident with higher Cx43 mRNA, and not Cx43 protein levels suggesting that BPA may cause a change in the recruitment of Cx43 into lipid rafts [339]. In contrast, in another study, treatment of COCs isolated from mouse preovulatory follicles with BPA (2.2 and 2200 nM BPA for 2 h) decreases GJIC in the COCs and do not modify GJIC gene (Cx43 and Cx37) and protein (Cx43) expression. These contradictory results may be explained by the different developmental stages of the follicles [340].

In the rat epithelium-derived BICRM1Rk cell line, which expresses Cx43, BPA inhibits GJIC in an irreversible and dose-dependent manner (from 0.1 to 3.2 µM, for 1 h incubation). Moreover, BPA inhibits GJIC through a modulation of the gating of GJ channels and not through a modulation of Cx43 gene expression [322]. An electrophysiological study showed that BPA reversibly inhibits (10 nM, up to 18% of control) the outward currents of Cx46 HCs expressed in Xenopus oocytes and it was proposed that the endocrine disruptor occupies pore regions of the HC and thus retards current flows [309]. BPA (0.01–1 µM for 48 h) significantly inhibits GJIC between human skin HaCaT cells and significantly downregulates Cx26 mRNA expression (0.1 µM BPA) partially through the ER pathway. In contrast, BPA does not influence Cx43 mRNA, Cx26 and Cx43 protein expression levels and Cx locations in HaCaT cells [319]. Interestingly, an analysis of the Cx43 structure showed the presence of a docking ‘pocket’ in Cx43-based connexon for estradiol-17b and BPA. These interactions with channel putative docking domains can possibly lead to the blockage of Cx43-based GJIC [310,311].

### 3.3. Phthalates

Phthalates are dialkyl or alkyl aryl ester derivatives of phthalic acid (1,2-benzenedicarboxylic acid). They are nonsteroidal endocrine-disrupting chemicals that have estrogenic activity and thus deleterious effects on hormonal balance [369]. They are used in a variety of products including cosmetic products and lotions, aerosol delivery agents, plasticizers and adhesives, flooring and medical tubing [370]. Humans can be exposed to phthalates through dermal absorption, ingestion, or inhalation of contaminated products [371]. Phthalates are lipid soluble, have less than 24 h half-lives, and do not accumulate appreciably in the body [372]. Phthalate metabolite levels can be measured in the urine, blood, breast milk, and meconium. A series of branched chain phthalate monoesters that contained an ethylalkyl moiety (8 h treatment at sublethal concentrations) inhibit GJIC between male B6C3F1 mouse hepatocytes whereas straight chain phthalates have no effect (Table 3) [341]. Di-isononyl phthalate (DINP) and related C7-C11 dialkyl phthalates were shown to inhibit GJIC in the liver of F344 male rats or B6C3F1 male mice [343]. A further study using 8 phthalate monoesters [mono-2-ethylhexyl phthalate (MEHP), mono-n-octyl phthalate (MNOP), mono-isononyl phthalate (MINP, 3 types, -1, -2, and -3), mono-isoheptyl phthalate (MIHP), mono-isodecyl phthalate (MIDP), and mono-(heptyl, nonyl, undecyl) phthalate (M711P)] provides evidence that the effects of phthalate esters on GJIC are species specific and may not be relevant for human beings. All eight monoesters significantly reduce GJIC in cultures of primary hepatocytes from rat and mouse but not from hamster, cynomolgus macaque and human [344]. A recent work shows that among 20 different phthalates, those with a medium-length side chain (3–6C) were the most potent inhibitors of GJIC and activators of MAPK1/2 in rat liver WB-F344 cells [326].

### 3.4. Others

Polychlorinated biphenyls (PCBs) and polybrominated biphenyls (PBBs) are industrial chemical mixtures widely used commercially as hydraulic fluids, plasticizers, adhesives, heat transfer fluids, wax extenders, dedusting agents, organic dilutents/extenders, lubricants and as dielectric fluids in capacitors and transformers (PCB), and as a flame retardant (PCB and PBB). These compounds are believed to be endocrine-disrupting chemicals in humans and animals. PCB and PBB are persistent organic pollutants that have been widely detected in the environment including air, water, fish, wildlife, and also in human tissues [373]. The nonplanar PCB 153 (2, 2, 4, 4, 5, 5-hexachlorobiphenyl at 100 µM for 30 min exposure) completely inhibits GJIC in WB-F344 rat liver epithelial stem-like cells (Table 3) through the PC-PLC or sphingomyelinase and Src signaling pathways [327]. PCB 153 has no effect on Cx43 mRNA levels but induces Cx43 hyperphosphorylation, internalization of GJ plaques and degradation in rat liver epithelial cells [321]. PCB 153 inhibits GJIC through PC-PLC independent of Mek [324]. It was further demonstrated that PCB153 regulates GJIC and Cx43 expression in WB-F344 cells by SphK1/S1P axis [sphingosine kinase (SphK), the enzyme responsible for S1P (the bioactive sphingolipid (SL) sphingosine 1-phosphate (S1P) synthesis] [328]. Moreover, various derivatives of hydroxylated PCB inhibit GJIC in WB-F344 rat epithelial cells and have estrogenic activity [344]. PBB (7.5 µg/for 48 h) and several of its congeners significantly inhibit GJIC in V79 Chinese hamster lung cells in vitro [345,346]. As reported previously, at the non-cytotoxic concentrations of PCB and PBB congeners that inhibit GJIC, a reduction in the number of GJ plaques and in the Cx43 phosphorylation level were observed [313]. DCP (used in the manufacture of polymers and elastomers) and PFDA (used in the manufacture of Teflon and Gore-Tex) inhibit GJIC through PC-PLC independent of Mek whereas PFOA (used as surfactant and for the manufacture of polymers) requires both MEK1/2 and PC-PLC. In contrast, PFOS (a surfactant used to coat a wide range of consumer goods, specifically those designed to be waterproof) and 1-monolaurin (used as surfactant in cosmetics and as food additive) require neither MEK1/2 nor PC-PLC [324]. Several PAHs, a broad class of ubiquitous environmental pollutants, transiently inhibit in vitro GJIC between WB-F344 rat liver epithelial cells in the µM range concentrations [347]. Polycyclic aromatic hydrocarbons (PAHs) inhibit GJIC (Table 3) through PC-PLC independent of Mek [324]. Among them, 1-methylanthracene, a major component of the PAH fraction of cigarette smoke, inhibits GJIC in WB-F344 rat liver epithelial cells through a PC-PLC-dependent mechanism, and releases arachidonic acid [329]. TCDD, a dioxin released into the environment in the Seveso disaster, regulates the activity of GJIC in several cells (Table 3) [330,348,374]. TCDD decreases GJIC by phosphorylating Cx43 via PKC α signaling pathway in MCF-7 human breast cancer cells and by affecting the localization of Cx43 in human mammary epithelial cells (HMEC) [330]. AhR is a ligand-activated transcription factor activated by both endogenous and exogenous ligands including PCBs and PAHs. In epithelial WB-F344 cells, TCDD downregulates GJIC (Table 3), significantly decreases the amount of GJ plaques and reduces Cx43 protein levels, possibly via enhanced proteasomal degradation, in an AhR dependent manner [348]. Fine particles (PM_2.5_) refer to the airborne particles with aerodynamic diameters less than 2.5 µm, whose impact on public health has become of great concern in industrial countries [375]. PM_2.5_ collected during the dust and non-dust periods significantly inhibits in a dose-dependent manner GJIC between human lung fibroblasts (Table 3) [349]. In an RNA microarray assay using endothelial cells, PM_2.5_ induced a dysregulation in transcripts involved in GJ signaling pathways [376]. So far, no effect of airborne ultrafine particles (PM_0.1_; 1–100 nm diameter) has been reported on GJIC. However, the fact that such particles induce neurodevelopmental disorders in mice suggest that studies examining possible effects of ultrafine particles on GJIC in the brain context should be done [377]. A number of toxic metals, which are major environmental pollutants (e.g., mercury, lead, arsenic, cadmium and aluminum) have been shown to interfere with GJs. It was proposed that GJIC inhibition (Table 3) is a mechanism of toxicity of these metals involved in the onset of many diseases [350,351,378].

## 4. Pathologies

A dramatic example of developmental neurotoxicity due to pollution was the severe methylmercury poisoning which occurred in Minamata and neighboring communities in Japan during the 1950s and 1960s. Afterwards, a considerable number of children were born with conditions resembling cerebral palsy, later known as congenital Minamata disease [379]. This was an acute example showing that environmental pollution can affect CNS development. From then, the prevalence of increasing brain pathologies over the last decade might also be the consequence, at least partly, of environmental pollution (Table 4). Recent evidence suggest air pollution as a source of neurodevelopmental toxicants [380,381]. Interestingly, it seems that these effects may depend on the type of exposure. This is what tends to be revealed in a PubMed search, which found associations between short-term air pollution exposure with AD, epilepsy, and migraine, while long-term air pollution exposure was associated with AD, whereas evidence on the link between air pollution and PD was inconsistent [382]. Despite conflicting results, children exposed to air pollutants (i.e., ambient or traffic-related air pollution, PAHs, and black carbon) during prenatal and postnatal periods seem to be at greater risk of cognitive deficits, behavioral disorders, and psychiatric disorders [383,384,385,386,387,388,389,390,391]. Children are indeed particularly vulnerable to exposure, prenatally and in early life when organ systems, including the brain, are under development. Therefore, for example, ASD now affects up to 1 in 45 children in the USA [392]. A total of 11% of children are affected by ADHD, another neurodevelopmental pathology, and a decline in child intelligence quotient was observed amongst Western nations [393]. PM_2.5_ appears to be an especially harmful pollutant because it enters the circulation and reaches the brain directly via cerebral blood supply. Prenatal exposure to PM_2_._5_ has been associated with reductions in brain white matter volume, cognitive impairments, and increases in ADHD, migraine and MDD [394,395,396]. Moreover, air pollution exposure in urban residency was associated with adolescent psychotic experiences [397]. Acute and subclinical pesticide poisoning that occur during childhood might lead to lasting neurobehavioural deficits [398]. Environmental exposures during adult life can also increase the risk of neuropathies. This is particularly the case for occupation-related exposures as farmers or even workers exposed to welding fumes (WFs), which comprise a complex mixture of metallic oxides, silicates, and fluorides, resulting in different health effects. Inhalation of WFs in large quantities over long periods may pose a risk of developing neurodegenerative diseases (PD, AD, and epilepsy) by dysregulating gene expression [399].

Emerging evidence suggested that alterations in normal astrocyte function contributes to the etiology and progression of varied neurological disorders including migraine with aura, neurodegenerative disorders, schizophrenia, epilepsy, and ASD. Therefore, targeting astrocyte dysfunction may lead to new therapeutic strategies for these disorders [450,451]. On the other hand, GJs and in particular Cx43 GJs, the predominant Cx in astrocytes, have emerged as a target for many environmental pollutants. One open question is whether pollutants by acting on Cx43 GJs in the astroglial network, or other Cxs of other brain GJ compartments, can induce cerebral neuropathies and cancer.

### 4.1. Neurodevelopmental Disorders

#### 4.1.1. Autism Spectrum Disorders

ASD is a neurodevelopmental disorder characterized by deficient social interaction, impaired communication as well as repetitive behaviors. ASD subjects present connectivity and neuroplasticity disturbances associated with morphological alterations in axons, dendrites, and dendritic spines. The etiology of ASD is believed to be multifactorial and to involve genetic, epigenetic, and environmental components [398]. Environmental chemical exposures are increasingly understood to be important in causing neurotoxicity in foetuses and newborns [452,453]. Studies using conditional Cx43 KO mice show that Cx43 is important for neurodevelopment [135]. However, few data are available about the precise involvement of GJs and HCs in ASD (Table 4) [406,454].

Neuroanatomical studies have revealed extensive structural abnormalities in subjects with autism as well as evidence of neuroinflammation. In post-mortem brain tissues, Cx43 protein expression (western blot) was increased significantly in superior frontal cortex (Broadmann’s area 9) [406], an area where structural abnormalities have been revealed in subjects with autism [455]. Dysfunction in this region may be responsible for deficiencies in cognition, language, and social function [456]. Even if non-significant (54%), increased Cx43 was also observed in the parietal brain region (Broadmann’s area 40) [406]. This increase with those of other astrocytic markers (aquaporin 4 and GFAP) may potentially contribute to dysfunctions observed in subjects with autism such as language and visuospatial integration [457,458]. Such increase in Cx43 expression in the brain may significantly increase glial-neuronal signaling, which is potentially responsible for GJIC enhancement in frontal lobe, an area for executive function. This may help to explain defects in sensory processing observed in subjects with autism [459,460].

It has been said that there is a strong correlation of general gastrointestinal disturbances with autism severity [461]. Therefore, it was hypothesized that loss of Cx43 expression in enteric glial cells contributes to brain inflammation in ASD by inducing disturbances in the gut-brain axis [462]. In another respect, acupuncture intervention of «Changqiang» (GV 1) improves learning-memory ability in autism rats, which may be related to its effects in up regulating expression levels as determined through immunostaining of Cx43, Cx32 and Cx36 in the frontal cortex [463].

It was suggested that perinatal mastocyte activation by infectious, stress-related, environmental, or allergic triggers, with a subsequent release of pro-inflammatory and neurotoxic molecules, can contribute to brain inflammation in ASD pathophysiology. Exposure to aberrant environmental conditions and to chemical contaminants may be a potential risk factor for ASD and is the subject of considerable current epidemiological studies and speculation [400,401,402,403]. Thus, autism susceptibility genes are selectively targeted by numerous environmental chemical pollutants (BPA, pesticides, heavy metals, PM, phthalates, etc.) and by many compounds used in food, cosmetics, or household products [404].

#### 4.1.2. Attention Deficit Hyperactivity Disorders

According to the National Institute of Mental Health, ADHD is a disorder marked by an ongoing pattern of inattention and/or hyperactivity-impulsivity that interferes with functioning or development. Frequently, patients have difficulty sustaining focus and are disorganized (inattention), move constantly, including in situations in which it is not appropriate (hyperactivity). Some people with ADHD only have problems with one of the behaviors, while others have both inattention and hyperactivity-impulsivity. Most children have the combined type of ADHD. However, in preschool, the most common ADHD symptom is hyperactivity. All these symptoms interfere with or reduce the quality of how they function socially. ADHD symptoms can appear as early as between the ages of 3 and 6 and can continue through adolescence and adulthood. ADHD symptoms can change over time as a person ages. In young children with ADHD, hyperactivity-impulsivity is the most predominant symptom. As a child reaches elementary school, the symptom of inattention may become more prominent and cause the child to struggle academically. In adolescence, hyperactivity seems to lessen but inattention, restlessness, and impulsivity tend to persist into adulthood. Factors contributing to ADHD are not well known (Table 4) but various exposures during pregnancy have been mentioned (cigarette smoking, environmental toxins, alcohol, or drug use), or exposure to high levels of lead at a young age [407,408,464]. Several epidemiological studies have demonstrated the linkage between exposure to high levels of air pollution and ADHD [409,410,411]. This assumption was even confirmed recently in childhood [412]. Additional studies also reported a particular role of exposure to the agricultural and combustion pollutant, nitrous oxide (N_2_O), which might be a primary environmental trigger in the onset of not only ADHD but also of other neurodevelopmental disorders, such as ASD. On this particular aspect of agricultural pollutants, glyphosate could also be a factor responsible for ADHD [413].

Genetic causes have also been suggested for ADHD, which seem to implicate candidate genes involved in processes like neuronal migration, cell adhesion and proliferation [414]. One of the possible genetic causes is 1q21.1 recurrent microdeletions linked to developmental delay, with 10–25% of carriers being ADHD patients. Among other brain-related symptoms, intellectual disability was observed in 25–50% of cases, mild-moderate developmental delay (speech and motor delays) in 50–75% of cases and autism less than 10% [465,466,467]. This chromosomal portion carries several genes (PRKAB2, CHD1L, BCL9) along with two Cx genes (GJA5 and GJA8) encoding Cx40 and Cx50. Those Cxs are known to be involved in eye disorders (cataracts, microophtalmia, glaucoma, …) for Cx50 and cardiac defects (atrial fibrillation and structural defects) for Cx40 but not in brain disorders so far (Table 4) [468,469,470,471,472,473,474].

#### 4.1.3. Epilepsy

Epilepsy is a neurological disorder affecting anyone, at any age (even if it is the most common neurological disease in children), and in which abnormal brain activity causes seizures (periods) of unusual behavior, sensations, and sometimes loss of awareness. Seizure symptoms vary widely from staring blankly for a few seconds to violent shakings and twitching of the body. Recently, seizures have been divided into focal, generalized, unknown onset, with subcategories of motor, non-motor, with retained or impaired awareness for focal seizures [475]. Those symptoms are the consequence of abnormal excessive or synchronous neuronal activity in the brain. Recurrent seizures can result in hippocampal damage and seriously impair learning and memory functions in children. In a rat model, this phenomenon was related to neuronal apoptosis [476]. In the past years, several studies showed that epilepsy could be associated with either short-term or long-term chemical environmental exposures occurring during pregnancy or any time after birth (Table 4).

If old studies reported that environmental chemical odors was associated with increased rates of particular forms of epilepsy [477], it became evident, more recently, that air pollution (gaseous and fine particulate air pollution) can be a risk factor for epilepsy as shown in various urban areas in Chile [415] or in China. In the latter country, severe air pollution (PM_10_) episodes were clearly associated with increased epilepsy rates [416,417] even, if curiously, cumulative exposure with NO_2_ and CO appeared protective [417]. However, another study, performed in the city of Xi’an, showed that NO_2_ and SO_2_ were positively associated with epilepsy [418]. In support of such observations, a growing number of studies have shown that exposures to air pollutants can activate cellular mechanisms involved in epilepsy [478].

Such correlations have been also observed in cases of chemical poisoning and intoxication. For instance, in Japan, domoic acid of diatoms poisoned people after consumption of contaminated mussels; some individuals had seizures such as temporal lobe epilepsy a year after the event [479]. Reports indicate that intoxication of humans with organotin compounds (Tributyltin, TBT) could also be associated with neurological symptoms such as epilepsy and amnesia, perhaps by affecting synaptogenesis and neuronal survival, particularly in early development [480]. Pollutants such as perfluorooctane sulfonate (PFOS) induce tonic convulsion in rodents when ultrasonic stimulus is applied [481,482]. In humans, developmental PCB exposure is known to increase the susceptibility to audiogenic seizures in adulthood [419,420]. The risk of epilepsy and certain types of cancer (cervical cancer) may be increased among adults who were exposed to perchloroethylene-contaminated drinking water during gestation and early childhood [421].

Apparently, lack of GJIC between astrocytes seems to be relevant to epileptogenesis (Table 4) [145,423] even if an increased presence of Cx43 has been observed in hippocampus of epileptic patients [424]. A consequence of such a situation might be the reduction of spatial buffering of coupled astrocytes leaving neurons more susceptible to microenvironmental changes, resulting in spontaneous epileptiform activity and reduced threshold for evoking seizure activity [47,48]. The inhibition of astrocyte coupling during the early phase of epileptogenesis is not caused by reduced amounts of Cxs, but rather, associated with changes in the phosphorylation status of Cx43.

### 4.2. Neurodegenerative Disorders

Causes of both PD and AD have not been elucidated and several endogenous (genetic) and exogenous (environment) factors contribute to the onset and/or development of these illnesses (Table 1).

#### 4.2.1. Parkinson’s Disease

PD is a chronic and progressive disabling neurodegenerative disorder characterized by the selective loss of dopaminergic neurons of the substantia nigra pars compacta, resulting in marked impairment of motor control, and the presence of cytoplasmic protein aggregates, known as Lewy bodies, in dopaminergic nerve cells. Few studies have linked GJs and Cxs to PD pathophysiology (for review: [483]). Cx43 upregulation has been identified in the striatum of rodent models of PD and in cultured astrocytes stimulated with rotenone [484]. Moreover, gastrodin, a constituent of a Chinese herbal medicine, ameliorates PD by downregulating astrocytic Cx43 [485]. α-synuclein, a component of Lewy bodies, enhances the opening of Cx43 HCs and Panx1 channels in mouse cortical astrocytes which might constitute a novel mechanism involved in the pathogenesis and progression of different α-synucleinopathies including PD [486].

The current view regarding PD etiology is that a genetic basis interacts with environmental factors, causing the disease (Table 1 and Table 4). A systematic review and meta-analysis up to 2018 found weak evidence for an association between air pollution, mostly originating from traffic, and PD [433]. Several studies have also suggested a relationship between BPA exposure and PD pathophysiology [435]. Similarly, epidemiological and experimental studies have emphasized the association between neurodevelopmental and neurodegenerative disorders and pre- and post-natal and chronic exposure to the pesticides [401]. Due to occupational exposure to pesticides, farmers and chemical workers are at increased risk of PD occurrence (long-term brain damage, cognitive dysfunction and some serious behavioral and neurodegenerative disorders). The exposure to neurotoxic metals (such as Pb, Hg, Al, Cd, Mn and As), to PCBs, and solvents have also been implicated in PD etiology [434,436]. Glutamate excitotoxicity is considered to be involved in neurodegenerative diseases such as PD and AD. A recent study suggested that Cx43 GJs are involved in glutamate excitotoxicity induced by Mn exposure. Elevated Mn exposure increases astrocyte apoptosis and significantly disruptes glutamate homeostasis, accompanied by GJIC inhibition and decreased expression of Cx43 [438].

#### 4.2.2. Alzheimer’s Disease

AD is the leading cause of dementia in USA and afflicted more than 5.7 million Americans in 2018 [487]. It is an age-related dementia whose progressive histopathological hallmarks are amyloid-β (Aβ) deposition, gliosis, neuronal death, and synaptic loss in brain areas like cortical and hippocampal regions (Table 1) [488]. The progression of the disease leads to loss of memory and language ability, as well as increased anxiety. Epidemiological reports also indicate that obesity and type 2 diabetes are important risk factors but pollution could also be involved [439,440,489]. Stroke history may also increase risk of developing AD, as it was observed in rats in which stroke is induced by striatal injection of endothelin-1 [490]. Deposition of Aβ (mainly Aβ 40 and 42) aggregates up to forming amyloid plaques associated with a reactive gliosis [488]. In those areas, activated microglia are recruited and reactive astrocytes exhibit a morphofunctional remodeling modifying their interactions with neurons [491]. Interestingly, this sequence of cellular events has been associated precisely with Cx43 expression and/or function in post-mortem brains of AD patients [492,493,494], and by the use of various models such as genetically-modified mice [441,495,496,497] or chemically-treated rats [498].

The early step of activated microglia leading to neuroinflammation seems to first involve mast cells (MCs). Mainly located in meningeal layers and in cerebral parenchyma lying between the BBB and astrocyte endfeet [499], these cells migrate towards Aβ deposits [500] even before plaque formation [496]. Aβ peptides appear to activate Cx43 HCs (and also Panx1) of MCs which mediates Ca^2+^ influx required for releasing proinflammatory mediators (histamine, ATP, cytokines) and recruiting other cells to the neuroinflammatory response [442,501,502]. Therefore, MCs by acting as early sensors of amyloid peptide would play a critical role in the onset of AD [442,503]. Then, the presence of activated microglia is a source of proinflammatory cytokines known to inhibit GJIC but activate Cx43 HCs in astrocytes [50,51,92,299,441]. Such activation leads to ATP and glutamate release with neurotoxic effects [443,444,503]. However, it has been shown that Aβ oligomers also contribute to Cx43 HC activation by dysregulating Ca^2+^ homeostasis in astrocytes, especially through increased expression of key components of Ca^2+^ signaling (mGluR5 receptors and IP3R1) [504,505]. [Ca^2+^]_i_ increase induces Cx43 HC activity which also provide a pathway for Ca^2+^ entry in astrocytes. Therefore, a vicious cycle may take place in astrocytes in which high [Ca^2+^]_i_ triggers Cx43 HC openings that allow Ca^2+^ entry contributing to maintain elevated [Ca^2+^]_i_. One consequence of this process is a chronic Cx43 HC activation leading to release of glutamate and ATP [441,506,507,508]. Such release even amplifies this circle by stimulating in an autocrine manner purinergic and glutamatergic metabotropic receptors that contribute also to maintain high [Ca^2+^]_i_ in astrocytes [509]. Release of gliotransmitters (ATP/glutamate) through Cx43 HCs results in opening of Cx36 HCs and Panx1 channels in neurons [92,496]. Consequent neuronal Ca^2+^ overload leads to numerous deleterious consequences including structural neuritic alterations and increased oxidative stress [510]. This stress triggers nearby amyloid plaques and propagates into the cell body leading to caspase-dependent death in some vulnerable cortical neurons [511]. In in vitro assays, the acute application of amyloid-β25–35 (Aβ25–35) increases the activity of Cx36 along with Panx1 channels leading to potential neuronal death [92].

It is interesting to note that monomeric Aβ42 (mAβ) can stimulate the release of the major antioxidant glutathione (GSH) from cultured astrocytes through expression of the ABCC1 transporter. The fact that aggregated Aβ (not mAβ) reduces induction of ABCC1 expression supports the hypothesis that in the early stage of AD, less aggregated Aβ increases GSH release from astrocytes (via ABCC1 transporters and Cx43 HCs) providing temporary protection from oxidative stress that promotes AD development [512]. In line with this observation, several studies indicate that Cx43 is first neuroprotective [441,513,514,515,516]. Therefore, the upregulation of Cx43, which is observed in AD brains, may start as a neuroprotective response to amyloid plaques and explain clinical studies showing increased neuronal activity in the hippocampus and cortex in non-demented individuals at high risk for AD or at presymptomatic stage of familial AD [517,518].

Recent transcriptomic analysis made from post-mortem brain samples showed Cx43 as a major regulator of the expression of AD risk factor genes [519]. From these studies, it appears that Cx43 may even contribute to opposing neuroprotective and neurotoxic phenotypes as shown in previous studies [441,497,513,514,515,516,520]. Indeed, apparently, Cx43 initially supports neuronal functions by elevating gene expression signatures for the glial neurosupportive functions. This is in agreement with enhanced neuronal activity, which is found at the early stage of AD [517,518,521]. However, chronic Cx43 gain of function could lead to detrimental effects on neurons [522,523,524,525]. The molecular mechanisms permitting Cx43 to control gene expression are not known yet but could be monitored through interacting proteins such as drebrin, for instance, whose expression is decreased in brain of AD patients [526].

Taken together, these studies demonstrate that Cx43 HCs are involved, in different cell types, during all progressive steps of the disease. Therefore, strategies that target astroglial Cx43 HC activity seem to be an interesting option as suggested by Cx43 deficiency or pharmacological blockade in mouse models of AD [441,497,527]. Supporting such a hypothesis, the well-known Cx inhibitor, carbenoxolone, is able to reverse Aβ-induced alterations in rat brains [528] but it lacks specificity since it is a general blocker of HC activity but also of GJIC, independent of the Cx/Panx type. More specific blockers of Cx43 HCs are emerging, such as boldine, an alkaloid from the boldo tree, which specifically inhibits HC activity in astrocytes and microglia and decreases neuronal damage in mice model of AD [497]. Synthetic and endogenous cannabinoids (WIN, 2-AG, methanandamide) also reduce the opening of astrocytic Cx43 HCs, abolish Aβ-induced release of glutamate and ATP, and reduce neuronal damage in hippocampal slices exposed to Aβ [529]. Of particular interest, all types of Cx43 blockade (pharmacological or by gene depletion) were also able to diminish AD-related gene expression which is controlled by Cx43 [519].

### 4.3. Neurobehavioral Disorders

#### 4.3.1. Migraines

A recent study contributed to the limited available evidence, showing that short-term exposure to five air pollutants from traffic combustion sources, including PM_2.5_, may trigger migraine (Table 4). This association is especially pronounced on high temperature days [425]. Cortical Spreading Depression (CSD), a transient disturbance in electroencephalographic activity characterized by a slow wave of neuronal and glial depolarization that self-propagates across the brain cortex or other brain areas, was suggested to trigger migraine aura and to be responsible for activation of the trigeminovascular system and possibly migraine headache [530]. GJ-mediated propagation of Ca^2+^ waves and Cx-HCs in astrocytes have been proposed to be involved in CSD propagation. Thus, GJ and HC blockade would represent a possible therapeutic strategy [426,531,532].

#### 4.3.2. Major Depressive Disorders

Despite progress in understanding the etiology and pathophysiology of depression, no established mechanism can currently explain all facets of the disease and many questions remain unresolved. The etiology of depression seems to be determined by multiple influences, including genetic, social, and environmental (Table 1) [394]. However, it is interesting to note that studies using animal models of depression as well as post-mortem analysis of brains of patients with MDD suggest GJIC dysfunction by astrocytic GJ and/or Cx43 HCs while antidepressants would correct these molecular abnormalities. GJs containing Cx43 decrease the propagation of spreading depression, which is a pathological event leading to neuronal inactivation, by facilitation of uptake of glutamate and K^+^ [57]. These data suggest a link between the expression/function of Cx43 and depressive disorders, thus paving the way for a better understanding of its physiopathology and new therapeutic targets (Table 4) [432,533]. Some environmental pollutants (BPA, phthalates, heavy metals, pesticides, airborne pollutants emitted from vehicles, industries, etc.) are endocrine disrupting substances, which may increase the risk of depression, especially among susceptible individuals. Several studies suggest that prolonged exposure to BPA would have neurotoxic effects and would impact human brain development. Such exposures would then contribute to an increased prevalence of behavioral disorders and neuropathies (Table 4). A relation between prenatal and childhood BPA exposure and depressive symptoms among children, with a more pronounced effects among boys, was proposed in several studies [427,430]. In rodent models, offspring gestationally and/or lactationally exposed to BPA have also been reported to exhibit comparable behavioral deficits [534]. As adults, F1 male offspring of mouse dams exposed to BPA doses representative of human exposure levels, from preconception until lactation, exhibit increased depressive-like behavior [535]. Interestingly, developmental exposure of mice to BPA is associated with impaired synaptogenesis and spinogenesis, two estrogen-mediated processes that can persist into adulthood [536]. Recent epidemiological studies show associations between urinary phthalate levels and depressive symptoms among adult and elderly populations [395,431]. Some epidemiological studies also show an association between urinary heavy metals (Mn, Sn), PAHs, and depressive symptoms in adult population [431]. Exposure to heavy metals can occur through, for example, diet, traffic, and industrial emissions, including dust in industrial and urban areas. Other studies have suggested an association between Cd, Pb, Hg exposure and increased risk of depression [394]. Moreover, an increasing number of studies suggests an association between prenatal and adult pesticide exposure and risk of depression [394,401]. A positive association between the exposure to air pollution and an increase in the risk of depression was also demonstrated [394]. Among these studies, a recent exploratory analyse conducted on 284 London-based children concludes that children who grow up in a highly polluted environment are three to four times more likely to develop MDD by the age of 18. Such an observation suggests that PM_2.5_ enters the blood circulation, crosses the BBB, and induce brain inflammation, which is linked to depression [396].

### 4.4. Glioma

The most common brain tumours are gliomas in which the Cx situation is particularly complex (Table 4). Apparently, Cx expression globally decreases during the progression of these tumors. This is the case of Cx43, which has been the most studied Cx due to its high expression in astrocytes from which gliomas are thought to derive [537]. Experimentally, it has been shown that Cx43 expression is inversely correlated to cell proliferation [538,539] probably through its interaction with src [540]. However, glioma cells still expressing Cx43 have been shown to be more invasive either by interacting with surrounding astrocytes [541,542], establishing GJIC with other glioma cells [543] or through its C-terminal able to interact with actin cytoskeleton [544,545,546]. Therefore, Cx43 expression would be involved in glioma invasion. Nevertheless, the situation is even more complicated by the cellular heterogeneity of gliomas during their progression in which cancer stem cells were shown to express Cx46, which is also involved in the aggressive phenotype of those tumorigenic cells [547]. Globally, the Cx involvement in glioma is complex and is not simply a consequence of lack of expression of Cx43 [548]. The expression of Cx changes during progression and, according to the cancer cell type (stem or not), has different effects on proliferation and/or invasion [549]. At this time, it is not known why and how these regulations of Cx expression do occur during glioma progression.

The incidence of CNS tumor is increased in some European countries. Several environmental exposures have been investigated as potential risk factors, but for most, scientific evidence is still lacking. The role of pesticides in CNS tumor risk, especially gliomas and meningiomas, was first investigated by studies of mortality rates in farmers in the USA or Europe but results do not all converge [445]. Previous reviews have concluded that there was a weak-positive association between brain cancers and farming [446]. In 2013, a meta-analysis supports an association between parental occupational exposure to pesticides or residential exposures to pesticides and childhood brain tumors [447,448]. However, a meta-analysis in 2015 indicated that pesticide exposure and the risk of adult glioma have no association [550]. Interestingly, a study shows that endocrine disrupters like endosulfan (an organochlorine insecticide) or 2, 4, 5 hexachlorobiphenyl (a PCB) inhibit GJIC and Cx43 expression in a dose-dependent manner in neuronal stem cells (Table 4). Knowing that those cells are involved in tissue homeostasis, such a disruption may act as a starting point of carcinogenesis [449].

## 5. Discussion

This review attempts to establish a link between the higher incidence of brain pathologies [1] and dysfunctions with the chronic exposure to pollutants. We hypothesize that the common link would be Cx function (GJIC and HC) which is altered both in brain disorders (Table 4) and by a wide range of pollutants (Table 3).

During the last decades, the brain has become a main focus for research motivated by the prevalence of a large emerging spectrum of brain pathologies [1]. These brain disorders are quite heterogeneous in their symptoms and the age of the affected patients. Some of them, affecting children, are probably the consequence of cerebral developmental defects while others, because of degenerative processes, affect elder people. Depending on the age at diagnosis and their etiology, these brain pathologies can be categorized as neurodevelopmental (ASD, ADHD, epilepsy), neurobehavioral (migraines, MDD) and neurodegenerative (PD, AD) disorders. Brain cancers, mostly represented by tumours of glial origin, can be considered as an extra category. In addition to the increased incidence of brain pathologies, some data report a general decrease in the intellectual quotient among children [393,551].

On the other hand, these last decades have also been characterized by an unprecedented accumulation of pollutants (pesticides, heavy metals, airborne particles, etc.) in the environment due to agricultural, industrial, and urban activities. Such an accumulation started from the 1950s and mostly concerns stable chemical compounds, which persist as permanent pollutants contaminating soils, water, and air. All around the world, for years and even through generations, such a large environmental pollution exposed wide parts of populations in large cities, industrial suburbs, and intensive agricultural areas. Even if health consequences of such long-term exposures are still probably underestimated, some recent studies reported that brain disorders could have, at least in part, environmental causes (Table 1 and Table 4). These observations are in line with the detection of environmental pollutants (pesticides, BPA, …) in human tissues [552,553] and biological fluids such as blood [554,555,556], urine [554,555,556,557,558,559] and maternal milk [560]. The brain is a putative target of chemical pollutants whose lipophilic properties permit them to cross the BBB [306,307] and, thus, reach the brain parenchyma (Figure 2). BPA, for instance, has been detected in human adult brains (0.91 µg/kg) [561] and amniotic fluid (8.3 ng/mL) [556] and urine from premature infants [562]. Knowing the possible deleterious effects of such pollutants, they are in a position to affect brain development and function, as has been recently observed through the impact on intellectual capacities of exposed children [563,564,565].

All of this leads to a crucial question, which is: what would be the effects of the presence of such pollutants on brain functions during prenatal development and from childhood up to adult life? Answering to this question is not a simple task because the deleterious consequences of chemical pollutants may go through a variety of pathways depending on their structure and physical properties. However, taking into account that GJs are both very present in brain (Figure 2, Table 2) and highly sensitive to a wide range of xenobiotics and carcinogens (Table 3), these channels could be, at least partly, a possible answer to the question. Indeed, many pollutants have long been reported to be inhibitors of GJIC [304,305] up to the point that GJIC assays were postulated to be used for screening such xenobiotics [566]. In vitro, almost all the pollutants inhibit GJIC suggesting a non-specific effect through a modification of membrane fluidity acting on the GJ gating. However, for several pollutants, an action through non-classical ERs and several intracellular signaling pathways were found to be involved in GJIC dysregulation. A seductive hypothesis, in view of the emerging role of Cx channels as major targets of pollutants, is that these channels are sensors of environmental and body contamination.

Considering the prenatal situation, exposure of developmental brain to pollutants can significantly induce damaging effects before the completion of BBB maturation, which only happens during the second postnatal year. Because BBB is immature, chemical pollutants are thought to easily enter the brain parenchyma and directly exert their toxic effects on neural tissue [307]. Indeed, many studies highlight the susceptibility of neurodevelopment to environmental exposures and its potential link to the genesis of cerebral pathologies. This hypothesis is known as the “developmental origin of health and disease” (DOHaD) hypothesis [567]. The first 1000 days of life between conception and the second postnatal year are a vulnerable period of human development [568]. This represents a window of very rapid growth and development and, in turn, of greater susceptibility to infection and sensitivity to chemical pollutants. During this period, the human brain undergoes significant changes involving cell proliferation and migration, synaptogenesis, and synaptic pruning [569]. Any exposure to environmental pollutants, interfering with GJIC during this window, could therefore be the source of a silent pandemic of neurodevelopmental toxicity. For instance, such exposures could interfere not only with the brain GJ compartments in formation but also with differentiation processes in which Cxs are involved (myelination, cell migration, etc.) (Figure 1, Figure 2 and Figure 3). Moreover, in late development, neuronal activity is susceptible to be highly affected because of their connections, which are mostly due to Cx36-made electrical synapses before birth. Any GJIC disturbance at this developmental period could then possibly induce alterations at the origin of brain developmental disorders as it has been observed when GJs are inhibited during embryogenesis [106].

From childhood up to adult life, it is conceivable that chemical pollutants may have deleterious effects on brain physiology by disturbing GJIC inside the different GJ compartments (neuronal, panglial syncytium and vascular; Figure 2; Table 2). As shown in Table 3, most studies reporting pollutants as GJIC inhibitors were carried out on cell models expressing Cx43 which is typically the most largely expressed Cx in brain, mostly by astrocytes and endothelial cells (Table 2; Figure 2). Therefore, based on these extensive in vitro data, Cx43 could be a major target of chemical pollutants depending on the condition that sufficient doses would be reached to inhibit its channel function. The pollutants in the blood circulation first impact Cx43 GJs of endothelial cells. Even if the precise role of Cx43 in BBB permeability is not completely defined yet, it is possible that disturbance of Cx43-mediated GJIC between endothelial cells would perturb BBB permeability. An immediate effect of such a permeability disturbance would be then a higher accessibility and exposure of brain parenchyma to circulating pollutants. Consequently, the panglial syncytium appears as a second putative target whose Cx43-mediated GJIC could be affected by the increased permeability to pollutants (Figure 2). Such a disturbance would lead to altered glucose feeding of neurons and other cells of brain parenchyma (distant astrocytes, oligodendrocytes, microglia, ependymocytes). Alteration of K^+^ siphoning from the extracellular space would also be an additional consequence of the affected panglial syncytium. Then, such alterations could perturb, in turn, neuronal activity, which is very sensitive to both glucose and extracellular K^+^ concentration variations. Deduced from the GJ compartmental organization, another possible effect of GJIC disturbance inside the panglial syncytium would be myelination defects affecting oligodendrocytes (Figure 2) and, thus, decreased velocity of action potentials along axons. Theoretically, all these GJIC effects are likely to have deleterious consequences on brain functions locally and could explain the emergence of disorders described in this review. Therefore, the scenario of any accumulation of pollutants occurring inside brain parenchyma could explain the higher risk of diagnosis of disorders among old populations. This is in line with studies showing a link between pollutant exposures and the apparition of degenerative disorders like PD and AD (Table 4). Keeping in mind that stem cell niches are very close to blood capillaries and that GJs are involved in their proliferation and differentiation, any disturbance at this level might interfere with maintenance of the brain parenchyma, behavioral adaptation, neuron repair, and, in turn, might play a role in degenerative processes. In addition to the direct adverse effects on GJIC that are mentioned above, there are also possible indirect effects on Cx function that pollutants could cause. A situation during which such an indirect effect could happen is “sterile neuro-inflammation”, when exogenous chemicals or particles induce neuroinflammation through a pathogen-free activation of microglia [570]. Such an activation would then increase expression of Cx43 in microglia favoring the release of pro-inflammatory cytokines (IL-1β, TNF-α) through Cx43 HCs, which affects astrocytic GJIC, and, in turn, synaptic activity. Afterwards, neuroinflammation, as a progressive phenomenon, may lead to a loss of structure and/or functions of neurons. Of note, prior to neuron cell death, some events occur in glia, specifically “reactive gliosis” characterized by activated microglia and reactive astrocytes in which Cx HCs participate. Neuroinflammation is found in most brain pathologies and is indeed associated with Cx changes of expression and/or function [454]. This aspect is important to consider since, in brain disorders, comorbidities are frequently described and can be partially explained by a common etiology like neuroinflammation.

The fact that a correlation between GJIC inhibition by chemical pollutants and the rising incidence of some brain pathologies is not obvious could be explained by the Cx HC involvement. For example, in MDD, Cx43-mediated GJIC and Cx-HC activity exert different effects in their pathophysiology [533]. In agreement with this hypothesis is the reverse relationship that has been observed in astrocytes between GJIC establishment and HC activity [50]. Moreover, despite numerous clues, such a link is not definitively proved yet mostly because of a lack of appropriate models. For instance, the inhibition of GJIC by pollutants has been tested frequently on cell lines that are not from brain origin or even from human origin. More investigations are needed, based on human materials, to definitively focus on Cx function as a target of pollutants and as a possible cause of brain disorders. An obvious point would be to estimate the presence of pollutants in the cerebrospinal fluid and verify their effects on brain cell cultures. For this aspect, more adapted in vitro models are necessary through the reconstitution, for example, of brain parenchyma or as brain organoid cultures. The use of in vivo models may not be useful because of possible differences existing between rodents and human in brain development and organization in terms of Cx expression. However, even if the involvement of Cxs would be proved in the etiology of brain disorders, this statement could not bring currently any obvious therapeutical option. Indeed, there is no real possibility so far to increase GJIC in order to counteract its inhibition by chemical pollutants. Some peptides are known to interfere with Cx43 HCs, which are activated in microglia during neuroinflammation. However, so far, it is probable that those peptides would not act precisely enough in such a sensitive environment as brain parenchyma. Moreover, this review is focused on Cxs and not on Panxs, which have similar functions as Cx HCs. Targeting Cx HCs without targeting Panxs might have a weak effect. Concerning the etiology of brain disorders, it is probably important to consider also Panxs when Cx HCs are involved. To be complete, some studies have revealed cells inside brain parenchyma may communicate through alternative mechanisms. For instance, exosomes, microparticles or apoptotic bodies allow the transfer of gliotransmitters, organelles, DNA/RNA, proteins and pathogens between glial cells and neurons [571]. On the other hand, direct glia-to-neuron signaling not only takes place via GJs as it has been observed in a few cases [88,572,573] but also through long intercellular processes termed tunneling nanotubes (TNTs) [574]. The real involvement of such alternative intercellular communication pathways in brain disorders and its possible links with Cxs is an open field of investigation.

To conclude, if, in the future, Cxs are established as an important etiological part of brain disorders, they might not be a therapeutical target. Without such a targeted therapeutical solution, and in absence of limiting the presence of persisting pollutants in the environment, pre- and post-natal human brains risk being susceptible to still-increased incidence of brain disorders. However, a limitation of exposure can be applied, at least, to air pollution whose control makes it as a preventable risk for human health. In several countries, political actions aiming to reduce air pollution had rapid and dramatic repercussions on health outcomes and all-causes of mortality [575]. Unfortunately, despite preventable air pollution, the use of certain pesticides that have been banned for many years still persist in the soil and continue to pose a threat to public health. This is the case everywhere, even if some studies tend to show a decreased level of exposure in the last decade; for instance, in Denmark [576]. In some areas of the world, pollution-related brain disorders can be even further increased by malnutrition since several studies show that diabetes alters the BBB permeability [577]. Thus, it is probable that the incidence of brain disorders and other chronic diseases will increase for the next decades, especially if effective healthy alternatives for use on crops are not yet available.

## Figures and Tables

**Table 1:** Categories, diagnosis, prevalence, symptoms and causes of brain pathologies discussed in this review.

Brain Pathologies [References]	Age at Diagnosis (Average)	Prevalence (% of Indicated Population)	Symptoms	Probable Causes
**Neurodevelopmental**				
ADHD ^1^ [6,7]	9–10 years old (may appear as early as 3 years old)	8–12% (of children worldwide)	-Attention deficit -Hyperactivity -Impulsivity	-Genetics -Exposures at pregnancy and young age (cigarette smoking, alcohol and drug uses, lead, …) -Brain injuries
ASD ^2^ [8,9,10]	From 2 years old or earlier	1% (of total population) *Prevalence increasing globally over past 50 years*	-Lack of social interaction -Restricted interest -Repetitive behavior	-Genetics -Prenatal and postnatal exposures to environmental factors
Epilepsy [11]	Any age	0.4–1% (of total population)	-Recurrent and brief episodes of involuntary movements that may be partial or generalized	-Genetics -Brain damage from prenatal or perinatal causes -Head injury -Brain infection (meningitis, encephalitis or neurocysticercosis, …) -Brain tumour
**Neurodegenerative**				
Alzheimer’s disease [2,12]	65 years old	10% (at age 65 and older in US) *146.2% increase in deaths from 2000 to 2018*	-Decline in memory, language, problem- solving -Decline in performing everyday activities through judgment, reasoning, and other cognitive abilities	-Age -Genetics -Environmental factors (cigarette smoking, alcohol use, …)
Parkinson’s disease [13,14]	58 years old (1 patient/2)	1% (above 60 years)	-Movement disorder (tremor, rigidity and bradykinesia) -Depression, anxiety	-Age -Genetics (5–10% of cases) -Environmental factors (pesticide exposure, synthetic heroine use, etc.)
**Neurobehavioral**				
MDD ^3^ [15,16]	41.4 years	28.2% (life time prevalence)	-Persistent sad mood -Anxiety -Irritability -Loss of interest -Loss of energy -Fatigue	-Genetics -Major life change, trauma, stress -Physical illness, medications
Migraines [17]	Any age	12% (of total population)	-Recurrent attacks of moderate or severe headache lasting from 4 to 72 h	-Changes in hormonal levels in women -Circadian disruption -Genetics -Environmental factors (alcohol use, nitrates, carbon monoxide, etc…)
**Other**				
Cancer ^4^ [4]	Children and adults (45–70 years old)	0.004–0.008% (of total population)	-Headache -Vertigo -Vision trouble (depends on tumor location)	-Genetics -Therapeutic irradiation -Electromagnetic fields (?) -N-nitroso compounds (food) -Unknown

**^1^** Attention deficit hyperactivity disorders. **^2^** Autism spectrum disorders. **^3^** Major depressive disorders. **^4^** Glioma.

**Table 2:** Gap-junctional organization, connexin expression and functions in human adult brain. Stem cells are not included. Abbreviations: Astro., Astrocytes; BBB, Blood-brain barrier; CMT1X, X-linked Charcot-Marie-Tooth disease type 1; Compart., Compartments; Cx, Connexin; Ependym., Ependymocytes; Endo., Endothelial; GJ, Gap junction; GJIC, Gap-junctional intercellular communication; HC, Hemichannel; KO, Knock out mice; Neuroinflam., Neuroinflammation; NPC, Neuronal progenitor cells; ODDD, Oculodentodigital dysplasia; Oligo., Oligodendrocytes. References are indicated between brackets. In some cases, connexin function as GJIC and/or HC is questioned (?) because not proven yet [25,26,27,28,29,30,31,32,33,34,35,36,37,38,39,40,41,42,43,44,45,46,47,48,49,50,51,52,53,54,55,56,57,58,59,60,61,62,63,64,65,66,67,68,69,70,71,72,73,74,75,76,77,78,79,80,81,82,83,84,85,86,87,88,89,90,91,92,93,94,95,96,97,98].

GJ Compart. /GJ Networks	Cellular Components		Connexin Expression				Brain-Related Physiological Effects of Cx Deficiency in:	
	Cell Types	Major Cell Functions	Cx Types (Major Cxs)	Cx Functions		Principal Physiological Roles of Major Cxs	Mice (Cx Knock out)	Human (Cx Gene Defect)
				GJIC	HC			
Neuronal	Neurons	Action potential transmission	Cx36 [25,26,27] Cx31.1 [23,29] Cx45 [30,31,32] Cx57 [33,34,35]	+ [27]	+ [28]	Electrical synapses (no synaptic delay): -Synchronization of interconnected neurons [27] -Excitability limitation in inhibitory networks [27] -Role in learning and memory [27]	Single KO Cx36: -Impaired short- and long-term memories [36,37] -Reduced motor learning [36,37]	Cx36: -Juvenile myoclonic epilepsy [38,39]
Panglial syncytium	Astrocytes	-Component of neuronal synapses (tripartite synapses) -BBB component (astrocytic endfeet)	Cx43 [40] Cx30 [40] Cx26 [41]	+ [42] + [42] ? [43]	+ [44] ?	-Glucose/lactate diffusion from capillaries to neurons and panglial syncytium (oligo., ependym.) [45] -K^+^ diffusion from neuron periphery and oligo. to capillaries [46,47] -Synaptic clearing of neurotransmitters, diffusion and recycling towards neurons [45] -Modulation of neuronal activity [48] -BBB maturation [49] -Gliosis and modulation of neuron activity through Cx43 HC activated by IL-1β/TNF-α (neuroinflam.) [50,51] or Ca2^+^/K^+^ [52,53,54]. Such activation leads to ATP release leading to paracrine/GJIC-mediated IP_3_/Ca^2+^ wave propagation (gliosis) [55] and gliotransmitters (glutamate) release (synaptic modulation) [56]	Single KO Cx43 (astro.): -Accelerated hippocampal spreading depression and enhanced locomotory activity [57] -Increased exploratory activity [58] -Anxiolytic-like effect in open field [58] Cx30: -Reduced exploratory activity -Anxiogenic behavior [59] Double KO Cx43 (astro.)/x30 [60]: -Vacuolated oligo. -Edematous astrocytes -Myelination abnormalities -Spatial memory impair -Sensimotor impair	Cx43: ODDD patients (according to mutations) [61]: -White matter change -Psychomotor delay -Epilepsy -Language disorder
	Oligo.	-Axon myelination to facilitate propagation of action potentials	Cx2 9[62] Cx32 [63] Cx47 [63]	? [64] + [62,65,66,67] + [62,65,66,67]	? [68]	-Myelin integrity [69] -K^+^ buffering from neuron periphery to astrocytes [63,70]	Single KO Cx29: -No myelin defect [71] Cx32 [72]: -Enhanced intrinsic excitability of neurons -Myelination defects in neocortex -Dysfunction of inhibitory synaptic transmission Cx47 [66]: -No behavior abnormality -Vacuolation of nerve fibers -Myelination abnormality Double KO Cx32/Cx47 [73]: -Myelination abnormality -Astrogliosis -Microglia activation Cx30/Cx47 [74]: -Myelination defects -Severe motor impairment -Decreased number of oligodendrocytes -Microglia activation	Cx32: CMT1X patients: -Mostly peripheral neuropathy [75] -Encephalopathy linked to metabolic stress (white matter lesions) [76] Cx47: Pelizaeus-Merzbacher-like disease [77,78]: -Myelination abnormality -Impaired psychomotor development
	Ependym.	-Lining cerebral cavities -Restrictive barrier between cerebrospinal and interstitial fluids -Facilitate circulation of cerebrospinal fluid by ciliated cells	Cx43 [79]	+ [79]				
Vascular	Endo. cells	-Lining brain capillaries	Cx37 [80] Cx40 [80] Cx43 [80]	+ + +		-BBB integrity [81] -Maintenance and control of endothelial barrier function [81]		Cx37 polymorphism: -Ischemic stroke risk? [82]
	Pericytes	-Regulation of blood flow through contraction capacity	Cx37 [80] Cx40 [80] Cx43 [80]	+ + +		-Pericyte contraction? [83,84,85,86] -Maintenance of vascular homeostasis [81]		
Other	Microglia	-Inflammation control -Phagocyte micro-organisms and damaged cells	Cx32 [87] Cx36 [88] Cx43 [89] (When activated)	? ? [88] (With neurons when activated) + [90,91]	+ [87] + [92]	-Neuron death [87] -Control of NPC proliferation, differentiation and migration [93,94,95,96,97] -Exchange of antigenic peptides? [98]		

**Table 3:** Effects of environmental chemical pollutants on gap-junctional intercellular communication of various mammalian cell types. Abbreviations: GJIC, gap-junctional intercellular communication; I, inhibition of GJIC; A, activation of GJIC. The names of the cell lines tested are indicated between parentheses.

Pollutants	GJIC	Cell Lines and Animal Models	References
**Insecticides**			
**Organochlorine insecticides**			
**DDT ^1^**	I	Mammalian cells	[305,312,331]
		Human breast epithelial cells	[313]
		Rat liver epithelial cells (WB-F344)	[324]
**Dieldrin**	I	Mammalian cells	[305,332]
**Chlordane, endosulfan, heptachlor, aldrin**	I	Mammalian liver cells	[305,333,334]
**Methoxychlor**	I	Oviductal cells	[335]
		Rat liver epithelial cells (WB-F344)	[325]
**Lindane**	I	Chinese hamster lung cells (V79)	[312]
		Mouse hepatocytes (B6C3F1)	[314]
		Rat myometrial smooth muscle cells	[315]
		Rat liver epithelial cells (WB-F344)	[316]
		Murine Sertoli cells (42GPA9)	[317,336]
**Chlordecone**	I	Chinese hamster lung cells (V79)	[337]
		Human embryonic palatal mesenchymal cells	[338]
**Mirex**	I	Chinese hamster lung cells (V79)	[337]
**Toxaphene**			
	I	Human breast epithelial cells	[313]
**Herbicides**			
**Ioxynil**	I	Rat liver epithelial cells (IAR20)	[318]
**Alachlor**	I	Rat liver epithelial cells (WB-F344)	[324]
**Fungicides**			
**Vinclozolin**	I	Rat liver epithelial cells (WB-F344)	[325]
**Pentachlorophenol**	I	Rat liver epithelial cells (WB-F344)	[324]
**Bisphenol A**			
	I	Rat epithelial mammary cells (BICRM1Rk)	[322]
	I	Cx46 HCs expressed in *Xenopus* oocytes	[309]
	A	Rat cumulus cell-oocyte complex	[339]
	I	Mouse cumulus cell-oocyte complex	[340]
	I	Human keratinocytes (HaCaT)	[319]
**Phthalates**			
**Branched chain phthalate monoesters**	I	Mouse hepatocytes (B6C3F1)	[341]
**Straight chain phthalates**	None		
**DINP ^2^**	I	Rat liver epithelial cells (WB-F344) Mouse hepatocytes (B6C3F1)	[342]
**C7-C11 dialkyl phthalates**			
**8 phthalate monoesters**	I	Rat and mouse hepatocytes	[343]
	None	Hamster, cynomolgus and human hepatocytes	
**Medium-length side chain (3–6C)**	I	Rat liver epithelial cells (WB-F344)	[326]
**Others**			
**PCB153**	I	Rat liver epithelial cells (WB-F344)	[324,327,328]
**Derivatives of hydroxylated PCB ^3^**	I	Rat liver epithelial cells (WB-F344)	[344]
**PBB ^4^**	I	Chinese hamster lung cells (V79)	[345,346]
**DCP ^5^, PFDA ^6^, PFOA ^7^, PFOS ^8^, 1-monolaurin**	I	Rat liver epithelial cells (WB-F344)	[324]
**PAH ^9^**	I	Rat liver epithelial cells (WB-F344)	[329,347]
**TCDD ^10^**	I	Human breat cells (MCF-7 and HMEC)	[330]
	I	Rat liver epithelial cells (WB-F344)	[348]
**PM_2.5_^11^**	I	Human lung fibroblasts	[349]
**Aluminium**	I	Rat astroglial cells	[350]
**Mercury**	I	Rat proximal tubular cells	[351]

^1^ 1,1,1-trichloro-2,2-bis(4-chlorophenyl) ethane; ^2^ di-isononyl phthalate; ^3^ polychlorinated biphenyls; ^4^ polybrominated biphenyls; ^5^ dicumylperoxide; ^6^ perfluorodecanoic acid; ^7^ perfluorooctanoic acid; ^8^ perfluorooctane sulfonic acid; ^9^ polycyclic aromatic hydrocarbons; ^10^ 2, 3, 7, 8-tetrachlorodibenzo-p-dioxin; ^11^ fine particulate matter with aerodynamic diameter less than 2.5 µm.

**Table 4:** Major brain disorders. Putative environmental causes and connexin characteristics/involvements. References are indicated between brackets.

Brain Disorders	Pathologies	Putative Environmental Causes	Connexin Characteristics and/or Possible Involvements
**Neuro** **developmental**	Autism spectrum disorders	**Prenatal exposures to:** -Traffic-related air pollutants [400] -Organochlorine pesticides [401] -Heavy metals (Hg and Pb) -Organic pollutants (DDT, PBDEs, PCBs) -Phthalates, BPA [402,403,404] -Nitrous oxide (N_2_O) [405]	Increased Cx43 expression (superior frontal cortex and less significantly in parietal region) [406]
	Attention deficit hyperactivity disorders	**Prenatal exposures to:** -Cigarette smoke -Environmental toxins -Heavy metals (Pb) [407,408] -Air pollution [409,410,411,412] -Nitrous oxide [405] -Glyphosate [413]	**In 10–25% of cases:** 1q21.1 microdeletion (Cx40 and Cx50 gene loci?) [414]
	Epilepsy	-Air pollution (gaseous, fine particles) [415] -Air pollution (PM_10_) [416,417] -NO_2_ and SO_2_ pollution [418] -PCB (audiogenic seizures) [419,420] -PCE (in gestation, early childhood) [421]	Lack of GJIC between astrocytes [422,423] Increased Cx43 expression in hippocampus [424]
**Neurobehavioral**	Migraines	-Traffic air pollution -PM_2.5_ [425]	GJ-mediated Ca^2+^ waves and Cx43 HC activation in astrocytes [426]
	Major depressive disorders	**Prenatal and childhood exposure:** -BPA [427,428,429,430] Postnatal exposures: -Phthalates [415,431] -Heavy metals (Mn, Sn, Cd, Pb, Hg) [394,431] -PAHs [431] **Prenatal and postnatal exposures:** -Pesticides [394,395] -Air pollution (PM_2.5_) [396]	GJIC and Cx43 HC function in astrocytes [432]
**Neurodegenerative**	Parkinson disease	-Traffic air pollution [433,434] -BPA [435] -Pesticides [401,436] -Metals (Pb, Hg, Al, Cd, Mn, As) [434,436] -PCBs [436]	Cx43 upregulation in rodent striatum [437] GJIC inhibition and decreased expression of Cx43 among astrocytes (Mn exposure) [438]
	Alzheimer disease	-Air pollution [439,440]	Cx43 HC activation [441,442,443,444]
**Cancer**	Glioma	-Pesticides (?) [445,446,447,448]	GJIC inhibition and decreased Cx43 expression in neuronal stem cells [449]

## Abbreviations

Aβ: Amyloid β; AD: Alzheimer’s disease; ADHD: attention deficit hyperactivity disorders; AhR: aryl hydrocarbon receptor; ASD: autism spectrum disorders; BBB: blood brain barrier; BPA: bisphenol A; BV: blood vessel; C-ter: carboxyl terminus; [Ca^2+^]_e_: extracellular calcium concentration; [Ca^2+^]_i_: cytosolic calcium concentration; CNS: central nervous system; COCs: cumulus cell-oocyte complexes; CSD: Cortical Spreading Depression; CSF: cerebrospinal fluid; Cx: connexin; DCP: dicumylperoxide; DDD: dichlorodiphenyldichloroethane; DDE: dichlorodiphenyldichloroethylene; DDT: 1,1,1-trichloro-2,2-bis(4-chlorophenyl) ethane; DINP: di-isononyl phthalate; E2: 17-beta estradiol; ER: estrogen receptors; GJ: gap junction; GJIC: gap junctional intercellular coupling; HC: hemichannel; JC: junctional coupling; KO: knock out; LV: lateral ventricle; M711P: mono-(heptyl, nonyl, undecyl) phthalate; MCs: mast cells; MDD: major depressive disorders; MEHP: mono-2-ethylhexyl phthalate; MIDP: mono-isodecyl phthalate; MIHP: mono-isoheptyl phthalate; MINP: mono-isononyl phthalate; MNOP: mono-n-octyl phthalate; NPC: neural precursor cell; PAH: polycyclic aromatic hydrocarbons; Panx: Pannexin; PBB: polybrominated biphenyls; PCB: polychlorinated biphenyls; PC-PLC: phosphatidylcholine-specific phospholipase C; PD: Parkinson’s disease; PFDA: perfluorodecanoic acid; PFOA: perfluorooctanoic acid; PFOS: perfluorooctane sulfonic acid; PM_2.5_: fine particulate matter with aerodynamic diameters less than 2.5 µm; S1P: sphingosine 1-phosphate; SGZ: subgranular zone; SphK: sphingosine kinase; SVZ: subventricular zone; TCDD: 2, 3, 7, 8-tetrachlorodibenzo-p-dioxin; V_M_, membrane potential; VZ: ventricular zone, WF: welding fumes.
